# GATA-type transcriptional factor SpGAT1 interacts with SpMIG1 and promotes lipid accumulation in the oleaginous yeast $Saitozyma \ podzolica$ zwy-2-3

**DOI:** 10.1186/s13068-022-02177-z

**Published:** 2022-10-08

**Authors:** Yulu Ran, Hui Xu, Qingzhuoma Yang, Yi Xu, Huahao Yang, Dairong Qiao, Yi Cao

**Affiliations:** Microbiology and Metabolic Engineering key laboratory of Sichuan Province, College of Life Science, Sichuan University, Chengdu, Sichuan 610065 People’s Republic of China

**Keywords:** $Saitozyma \ podzolica$ zwy-2-3, C/N ratio, SpGAT1, SpMIG1, Lipid metabolism

## Abstract

**Background:**

In oleaginous yeast, nitrogen limitation is a critical parameter for lipid synthesis. GATA-family transcriptional factor GAT1, a member of the target of rapamycin (TOR) pathway and nitrogen catabolite repression (NCR), regulates nitrogen uptake and utilization. Therefore, it is significant to study the SpGAT1 regulatory mechanism of lipid metabolism for conversion of biomass to microbial oil in $Saitozyma \ podzolica$ zwy-2-3.

**Results:**

Compared with WT, $\Delta gat1$, and OE::*gat*1, the lipid yield of OE::*gat*1 increased markedly in the low carbon and nitrogen ratio (C/N ratio) mediums, while the lipid yield and residual sugar of $\Delta gat1$ decreased in the high C/N ratio medium. According to yeast two-hybrid assays, SpGAT1 interacted with SpMIG1, and its deletion drastically lowered *SpMIG*1 expression on the high C/N ratio medium. *MIG*1 deletion has been found in earlier research to affect glucose metabolic capacity, resulting in a prolonged lag period. Therefore, we speculated that SpGAT1 influenced glucose consumption rate across SpMIG1. Based on yeast one-hybrid assays and qRT-PCR analyses, SpGAT1 regulated the glyoxylate cycle genes *ICL*1, *ICL*2, and pyruvate bypass pathway gene *ACS*, irrespective of the C/N ratio. SpGAT1 also could bind to the *ACAT*2 promoter in the low C/N medium and induce sterol ester (SE) accumulation.

**Conclusion:**

Our findings indicated that SpGAT1 positively regulated lipid metabolism in *S*.*podzolica* zwy-2-3, but that its regulatory patterns varied depending on the C/N ratio. When the C/N ratio was high, SpGAT1 interacted with SpMIG1 to affect carbon absorption and utilization. SpGAT1 also stimulated lipid accumulation by regulating essential lipid anabolism genes. Our insights might spur more research into how nitrogen and carbon metabolism interact to regulate lipid metabolism.

**Supplementary Information:**

The online version contains supplementary material available at 10.1186/s13068-022-02177-z.

## Background

Because of the shortage of fossil fuels and the rising use of first-generation biofuels, renewable and sustainable alternatives [1], like microbial lipid, the third generation of biodiesel generated by oleaginous yeasts, need to be researched [2]. Conventional oleaginous yeasts (like $Lipomyces \ starkeyi$, $Rhodotorula \ toruloides$, $Yarrowia \ lipolytica$, etc.) have been applied extensively, but they still have drawbacks, for instance, restricted feedstock accessibility and yields that fall short of theoretical lipid yields [3, 4]. When compared to existing conventional species, non-conventional oleaginous yeasts have become appealing research materials due to their benefits of species diversity, high biomass and lipid production, good tolerance to hydrolysate inhibitors, excellent biodiesel characteristics, and economically lucrative bioprocesses [3, 5].

In recent years, $Saitozyma \ podzolica$, a non-conventional oleaginous yeast of basidiomycetous species, has been discovered [6–8]. $S.\ podzolica$ DSM 27192 could produce lipid and sugar acids from glucose or xylose, and its lipid accumulation is influenced by fermentation factors such as pH, temperature, and carbon sources concentration [7, 9]. To increase yields in $S.\ podzolica$ DSM 27192, many lipid extraction techniques also have been developed [10–12]. However, lipid metabolism mechanisms in $S.\ podzolica$ still have yet to be investigated. In conventional oleaginous yeasts, the lipid mechanism is usually considered to be that nitrogen limitation inhibits isocitrate dehydrogenase activity, raising cytoplasmic citric acid levels and boosting lipid formation in the high C/N ratio medium [13]. While even among closely related species and strains, there is enormous metabolic diversity like marked variances in xylose utilization, especially among non-conventional oleaginous yeasts [1]. Therefore, the focus of this study is on the mechanisms of carbon and nitrogen sources regulating lipid synthesis.

GATA-family transcriptional factor GAT1 plays a critical role in nitrogen sources response and utilization pathways, nitrogen catabolite repression (NCR) and target of rapamycin (TOR) pathway [14]. TOR pathway has been shown to regulate lipid metabolism in several studies [15]. For example, the mammalian TOR complex1 (mTORC1) modulates lipid homeostasis via regulating S6 kinase beta-1 (S6K) and transcription factor TEF3 [16]. Inhibition of the target of rapamycin complex 1 (TORC1) triggers lipid accumulation in fungi, and rapamycin (TORC1 inhibitor) therapy increases TAG accumulation in $S.\ cerevisiae$ and oleaginous microorganisms (like *Chlamydomonas* *reinhardtii*, $Cyanidioschyzon \ merolae$ , and *Trichosporon* *oleaginosus*) [17–20]. Both TORC1 inhibition and deletion of *GAT*1, *GLN*3, and *SIT*4 in the TOR pathway affect lipid droplet replenishment in $S.\ cereviase$ [17, 21]. In nitrogen-limited or non-preferred nitrogen source conditions, inhibited TORC1 interacts with Tap42-Sit4 complexes to dephosphorylate GAT1, and dephosphorylated GAT1 enters the nucleus to regulate NCR-related gene expression and improve nitrogen sources absorption [22]. Whereas, there is limited evidence that GAT1, a key nitrogen response protein, activates lipid metabolism-related genes directly.

Cell growth is linked to carbon and nitrogen metabolism. Carbon catabolite suppression (CCR) in yeast impacts carbon source uptake and utilization via the SNF1-MIG1 pathway [22]. The cross-talk between the TORC1 and SNF1-MIG1 pathways modulates cellular metabolic states [23]. During glucose replete growth, TORC1 is highly active to increase the anabolic process, while serine/threonine kinase AMP-activated protein kinase (AMPK, homolog protein of sucrose non-fermenting 1 (SNF1)) is relatively inactive. Upon glucose starvation, AMPK is activated to increase the catabolic process and inhibit mTORC1 [24, 25]. Deletion of the transcriptional repressor *MIG*1 also causes TOR pathway nutrient-sensing dysfunction in $Cryptococcus \ neoformans$ [26]. Currently, little research has focused on how the TOR and SNF1-MIG1 pathways interact to modulate lipid metabolism, however, several genes in two pathways impact oleaginous yeast lipid synthesis. For instance, *SNF*1 and *MIG*1 deletion both improve $Y.\ lipolytica$ lipid production [27, 28]. Active SNF1/AMPK could repress fatty acid and cholesterol synthesis by inactivating acetyl-coenzyme A carboxylase (ACC) and 3-hydroxy-3-methylglutaryl-CoA reductase (HMGR) activities [29]. The C/N ratio of the fermentation medium is the most important parameter for lipid formation [30]. Therefore, unraveling the relationship between carbon and nitrogen source absorption will help us better comprehend lipid accumulation physiology.

In the current study, we explored how the transcription factor SpGAT1 with SpMIG1 regulated lipid metabolism in the promising non-conventional oleaginous yeast strain $S.\ podzolica$ zwy-2-3. We tested biomass, lipid yield, and residual sugar in $S.\ podzolica$ zwy-2-3 WT, $\Delta gat1$, and OE::*gat*1 strains which were cultivated at varying C/N ratio mediums. Then, with yeast one-hybrid and two-hybrid assays, we investigated whether lipid metabolism was influenced by SpGAT1 and the cross-talk between nitrogen and carbon metabolism.

## Results

### Cloning, identification, and phylogenetic tree analysis of SpGAT1

**Fig. 1:**
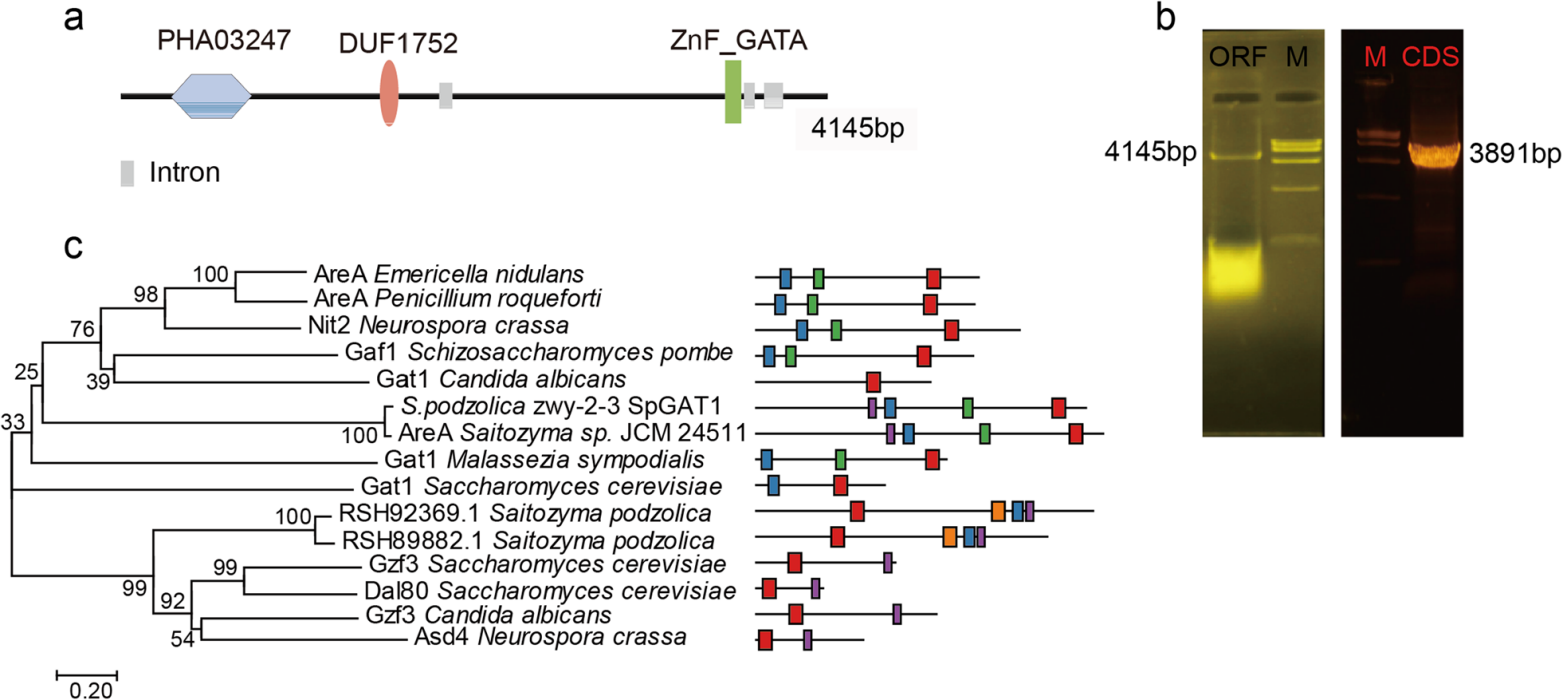
Identification of a GATA-type transcriptional activator SpGAT1 in $S.\ podzolica$ zwy-2-3. **a** Schematic representation of *SpGAT*1 gene structure. **b** Amplification of *SpGAT*1 ORF and CDS; left: lane 1:*SpGAT*1 ORF (4145 bp), lane 2: marker; right: lane 1: marker, lane 2: *SpGAT*1 CDS (3891 bp). **c** Phylogenetic analysis applying the Neighbor-Joining method and conserved domain analysis of SpGAT1 with other fungus species

**Fig. 2:**
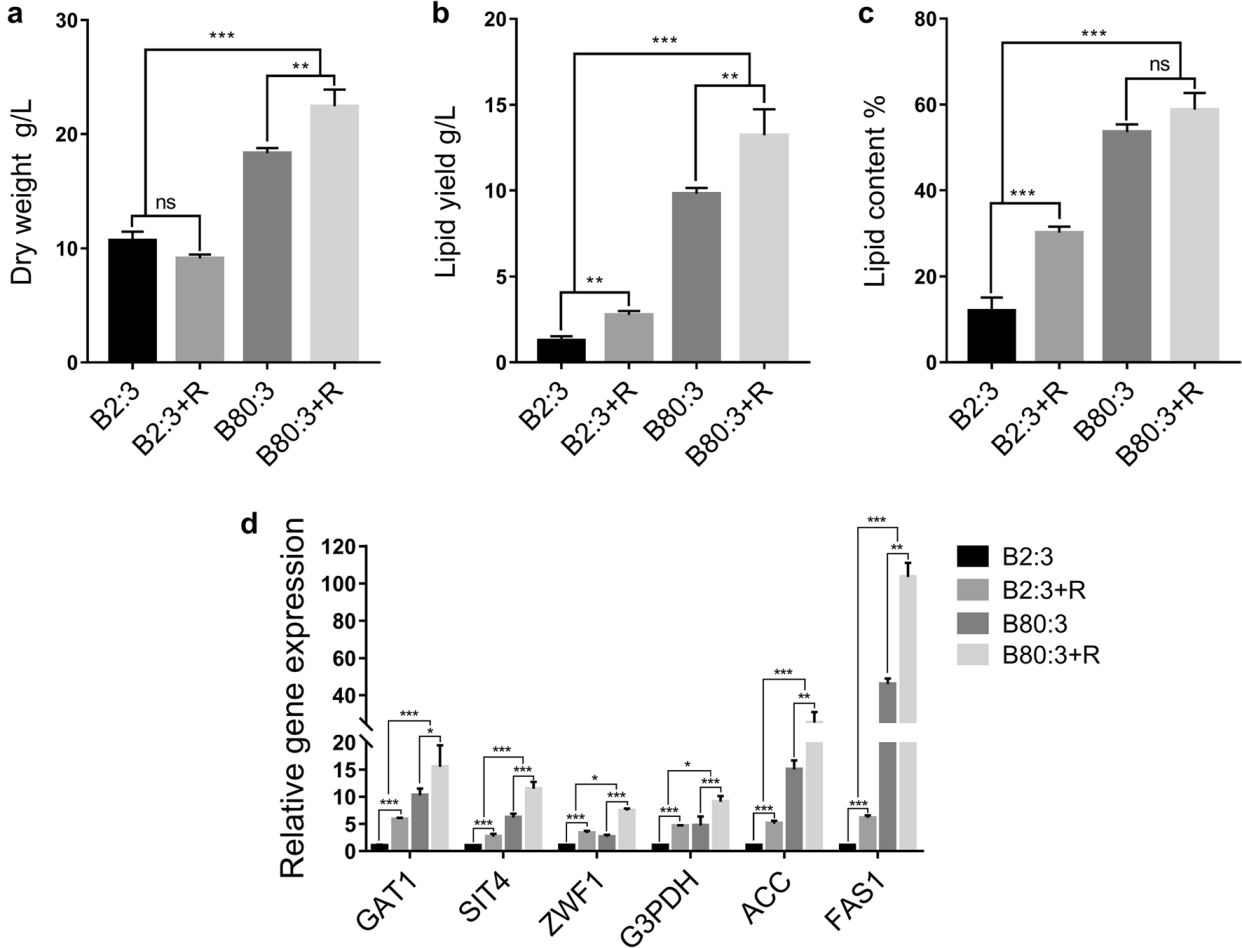
Cultivation of $S.\ podzolica$ zwy-2-3 in different C/N ratio (B2:3 and B80:3 groups) mediums which were co-treat with rapamycin. **a** Dry weight. **b** Lipid yield. **c** Lipid content. **d** Expression profiles of *GAT*1, *SIT*4, and lipid biosynthesis key genes, *GAT*1: encoding GATA-type transcriptional factor, *SIT*4: encoding phosphatase, dephosphorylate GAT1, *ZWF*1: encoding glucose-6-phosphate dehydrogenase, *G*3*PDH*: encoding glycerol-3-phosphate dehydrogenase, *ACC*: encoding acetyl-CoA carboxylase, *FAS*1: encoding fatty acid synthase 1. Data are presented as the means ( SE, $n = 3$). ns: no significant difference, *$p < 0.01$, **$p < 0.001$, ***$p < 0.0001$

SpGAT1 (GenBank accession number: RSH87488.1) was analyzed from the reference genome of $S.\ podzolica$ DSM27192 strain to identify SpGAT1 in $S.\ podzolica$ zwy-2-3. As shown in Fig. 1, SpGAT1 had two conserved domains, one of which was ZnF_GATA, a zinc finger DNA-binding domain (pfam: 00320), and the other of which was DUF1752 (pfam: 08550). SpGAT1 cloned from $S.\ podzolica$ zwy-2-3 was 4145 bp in length and contained three introns and four exons. SpGAT1’s CDS was 3891 bp (Fig. 1a, b), encoding 1297 amino acids with a predicted protein molecular weight of 135.23 kDa and an isoelectric point of 8.14. In addition, SpGAT1 was found to be most similar to AreA in $S.\ podzolica$ JCM 24511 after phylogenetic analysis (Fig. 2c).

### The TOR pathway and NCR enhance lipid accumulation through SpGAT1 in $S.\ podzolica$ zwy-2-3

To determine if the TOR pathway and NCR enhance $S.\ podzolica$ zwy-2-3 lipid production, we measured biomass and lipid yield in various C/N ratio mediums co-treated with rapamycin. Rapamycin treatment media (B2:3+R and B80:3+R) groups accumulated higher lipid than non-rapamycin treatment media (B2:3 and B80:3) groups. Low C/N media (B2:3 and B2:3+R) groups generated less lipid than high C/N ratio media (B80:3 and B80:3+R) groups. More specifically, after rapamycin treatment, the biomass of the B2:3 group decreased slightly from 10.69 g/L to 9.13 g/L, while the lipid yield increased significantly from 1.27 g/L to 2.76 g/L and the lipid content rose from 11.9 % to 30.2 %; the biomass of the B80:3 group increased from 18.33 g/L to 24.45 g/L and the lipid content increased little with rapamycin treatment, since the predominant lipid yield increase which increased significantly from 9.82 g/L to 13.22 g/L was attributed to the rising biomass (Fig. 2a–c). The results of single cell lipid droplets stained with Nile red matched with those extracted by the Folch method (Additional file 2: Fig. S1a). Fatty acid profiles in different groups did not differ significantly (Additional file 2: Fig. S1b). In conclusion, our results indicated that the TOR pathway and NCR contributed to lipid accumulation.

To further confirm SpGAT1’s role in the TOR pathway and NCR regulating lipid metabolism, we used qRT-PCR to analyze the transcription levels of *SpGAT*1, *SIT*4 (encoding PP2A-like protein phosphatase), and four genes encoding critical enzymes involved in lipid biosynthesis. *SpGAT*1 transcription level was strongly induced by rapamycin and nitrogen-limit treatment, and the relative expression levels of *SIT*4, *G*3*PDH* (encoding glycerol-3-phosphate dehydrogenase), *ZWF*1 (encoding glucose-6-phosphate dehydrogenase), *ACC* (encoding acetyl-CoA carboxylase), and *FAS*1 (encoding fatty acid synthase1) followed a similar expression trend with *SpGAT*1’s (Fig. 2d). Specifically, when rapamycin was treated, all gene expression levels in the B2:3+R group generally increased in comparison to the B2:3 group. For example, *SpGAT*1 and *SIT*4 relative expression levels increased by 6 and 2.7 times, respectively; *FAS*1 and *ACC* increased by 6 and 5 times, respectively; *ZWF*1, which provides NADPH for lipid synthesis, increased by 3 times. In contrast with the B80:3 group, *SpGAT*1, *SIT*4, *FAS*1, *ACC*1, and *ZWF*1 relative expression levels in the B80:3+R group increased by 1.5, 2, 2.2, 1.7, and 7.4 times, respectively. In nitrogen-limit treatment groups, *SpGAT*1, *SIT*4 and *ZWF*1 relative expression levels were 10, 6, and 2.6 times higher than those in the B2:3 group, respectively. Strikingly, the relative expression levels of *FAS*1 and *ACC*1 in the B80:3 group were 46 and 15 times higher than in the B2:3 group, respectively. These results suggested that SpGAT1 might be activated by the TOR pathway and NCR to regulate lipid metabolism of $S.\ podzolica$ zwy-2-3.

### SpGAT1’s effects on lipid accumulation and the uptake and utilization of various carbon and nitrogen sources

To perform function analysis of SpGAT1 in $S.\ podzolica$ zwy-2-3, a SpGAT1-knockout strain ($\Delta gat1$) and an overexpression strain (OE::*gat*1) were constructed. In the $\Delta gat1$ strain, *SpGAT*1 cannot be amplified by PCR and hygromycin had been inserted in the genome (Additional file 2: Fig. S2a,b). qRT-PCR validated the OE::*gat*1 strain, and its *SpGAT*1 transcription level was enhanced sixfold on the low C/N ratio medium compared to WT (Additional file 2: Fig. S2c, d).

**Fig. 3:**
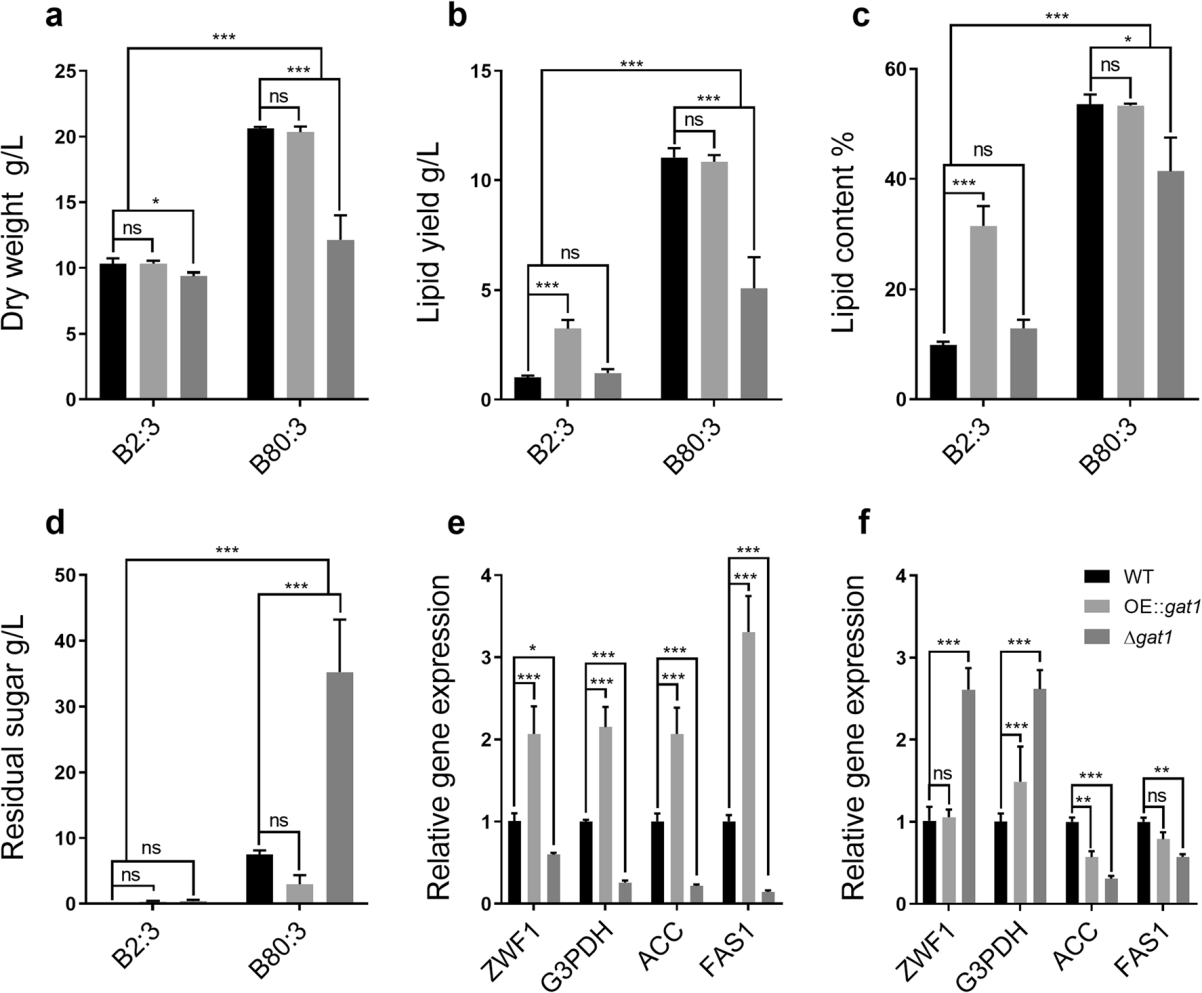
Effects of *SpGAT*1 mutants on cell growth and lipid biosynthesis in shake flasks. **a** Dry weight of WT, OE::*gat*1, and $\Delta gat1$ in the B2:3 and B80:3 groups. **b** Lipid yield of WT, OE::*gat*1, and $\Delta gat1$ in the B2:3 and B80:3 groups. **c** Lipid content of WT, OE::*gat*1, and $\Delta gat1$ in the B2:3 and B80:3 groups. **d** Residual glucose concentration in liquid supernatant of WT, OE::*gat*1, and $\Delta gat1$ in the B2:3 and B80:3 groups. **e** Expression profiles of *ZWF*1, *G*3*PDH*, *ACC*, and *FAS*1 of WT, OE::*gat*1, and $\Delta gat1$ in the B2:3 group. **f** Expression profiles of *ZWF*1, *G*3*PDH*, *ACC*, and *FAS*1 of WT, OE::*gat*1, and $\Delta gat1$ in the B80:3 group. *ZWF*1: encoding glucose-6-phosphate dehydrogenase, *G*3*PDH*: encoding glycerol-3-phosphate dehydrogenase, *ACC*: encoding acetyl-CoA carboxylase, *FAS*1: encoding fatty acid synthase 1. Data are presented as the means ( SE, $n = 3$). ns: no significant difference, *$p < 0.01$, **$p < 0.001$, ***$p < 0.0001$

Then, in different C/N ratio mediums, we analyzed the biomass, lipid yield, and residual sugar of WT, $\Delta gat1$, and OE::*gat*1 to explore whether SpGAT1 had impacts on $S.\ podzolica$ zwy-2-3 lipid metabolism (Fig. 3). In the B2:3 group, overexpression of *SpGAT*1 reinforced $S.\ podzolica$ zwy-2-3 lipid accumulation. Because the biomass and glucose consumption rate of $S.\ podzolica$ zwy-2-3 were dramatically decreased when *SpGAT*1 was knocked out, the B80:3 group’s lipid yield was drastically reduced. The biomass of WT, OE::*gat*1, and $\Delta gat1$ in the B2:3 group did not vary significantly, as shown in Fig. 3a. The biomass of $\Delta gat1$ declined from 20.6 g/L to 12.1 g/L in the B80:3 group as compared to WT, while OE::*gat*1 did not alter appreciably. SpGAT1 had distinct effects on lipid yield in media with high and low C/N ratios (Fig. 3b, c). The lipid yields of WT and $\Delta gat1$ in the B2:3 group were 1.02 g/L and 1.22 g/L, respectively, with no significant difference; however, the lipid yield of OE::*gat*1 soared to 3.24 g/L, and the lipid content increased from 9.9 % to 31.5 %. Unlike the B2:3 group, the lipid yields of WT, OE::*gat*1, and $\Delta gat1$, respectively, were 11.05 g/L, 10.84 g/L, and 5.08 g/L, lipid content of $\Delta gat1$ declined obviously from 53.6 % to 41.4 %, while overexpression *SpGAT*1 had no significant impact on lipid accumulation in B80:3 group. Interestingly, the glucose consumption rates of the three strains were clearly dissimilar (Fig. 3d). The three stains all most exhausted glucose in the low C/N ratio medium; while in the high C/N ratio medium, residual glucose of WT was approximately 7.5g/L and OE::*gat*1 was about 2.98 g/L left, and glucose consumption rate of $\Delta gat1$, which had residual sugar of around 35.22 g/L, was much lower than WT and OE::*gat*1. In addition, like rapamycin and nitrogen-limit treatment, fatty acid profiles of *SpGAT*1 mutants revealed no significant differences with WT (Additional file 2: Fig. S3a).

To further clarify how SpGAT1 impacts lipid metabolism, we analyzed relative expression levels of *FAS*1, *ZWF*1, *G*3*PDH*, and *ACC* in different C/N ratio mediums. SpGAT1 had a wide affect on several crucial lipid synthesis genes, as shown in Fig. 3e,f. In the B2:3 group, compared to WT, the transcription levels of *FAS*1, *G*3*PDH*, *ZWF*1, and *ACC* of OE::*gat*1 were significantly up-regulated 3.3, 2.1, 2.1, and 2.1 times, respectively; while the expression levels of these genes of $\Delta gat1$ versus WT were decreased by 0.14-, 0.26-, 0.59- and 0.22-fold, respectively (Fig. 3e). Except for transcription levels of *FAS*1 and *ACC* which were decreased by 0.57- and 0.31-fold, respectively. Lipid synthesis gene expression levels of $\Delta gat1$ in the B80:3 group exhibited only a slight change (Fig. 3f). qRT-PCR results were following the phenotypes of lipid yield. Therefore, these results revealed that SpGAT1, as a transcription factor, could regulate lipid metabolism and the glucose consumption rate of $S.\ podzolica$ zwy-2-3.

The capacities of the WT, $\Delta gat1$, and OE::*gat*1 growing on various nitrogen and carbon sources were assessed to further analyze the function of SpGAT1 in nitrogen and carbon absorption, as shown in Additional file 2: Fig. S3. On the one hand, the effect of SpGAT1 on nitrogen absorption and uptake was related to the nitrogen sources. Three strains grew well in the presence of preferred amino acids such as glutamine, glutamic acid, or inorganic nitrogen. However, the $\Delta gat1$ strain grew worse on non-preferred amino acids including serine, proline, methionine, tryptophan, isoleucine, and histidine (Additional file 2: Fig. S3b). Despite this, the $\Delta gat1$ strain grew more quickly on arginine than the other two strains. On the other hand, there was no evident difference in the consumption rate of various carbon sources between OE::*gat*1 and WT; whereas the $\Delta gat1$ strain used glucose, sucrose, mannose, xylose, and xylan at the slowest rate among the three strains (Additional file 2: Fig. S3c). These results reflected that SpGAT1 affected the abilities of nitrogen and carbon uptake and *SpGAT*1 deletion might disrupt metabolism.

### SpGAT1 promotes lipid accumulation by positively regulating key genes in the pyruvate bypass pathway and glyoxylate shunt pathway

SpGAT1-GFP subcellular localization is shown to be cytoplasmic, nuclear–cytoplasmic, or nuclear in Additional file 2: Fig. S4. This discovery confirmed that SpGAT1 could distribute in the nucleus and bound to promoters to affect genes’ transcription and expression. Then we wondered whether SpGAT1 might directly activate the transcript-level of genes associated with lipid biosynthesis, so we applied R package TFBSTools to screen the whole genome for genes with one or more binding sites on their promoters. After GO rich and conserved domains analyses, we ultimately selected six genes (*ACS*, encoding acetyl-CoA synthetase; *ICL*1 and *ICL*2, encoding isocitrate lyase1 and 2; *ACAT*1, encoding acetyl-CoA acyltransferase 1; *ACAT*2, encoding acetyl-CoA acyltransferase 2; *PDC*, encoding pyruvate decarboxylase) which had impacts on lipid metabolism.

**Fig. 4:**
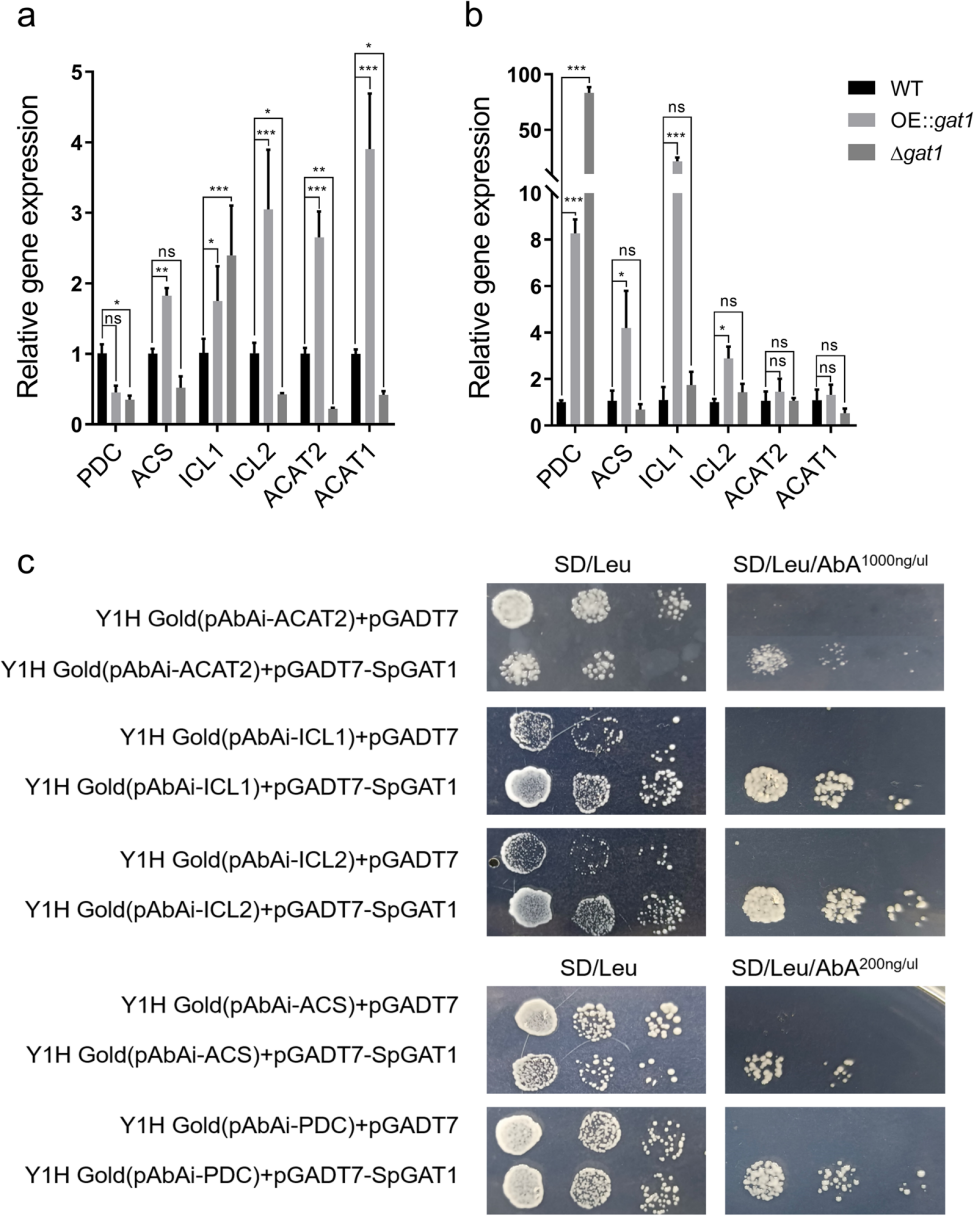
SpGAT1 directly bound to promoters of key genes in the pyruvate bypass pathway and the glyoxylate shunt pathway to promote lipid accumulation. **a** Expression profiles of *PDC*, *ACS*, *ICL*1, *ICL*2, *ACAT*2, and *ACAT*1 of WT, OE::*gat*1, and $\Delta gat1$ in the B2:3 group. **b** Expression profiles of *PDC*, *ACS*, *ICL*1, *ICL*2, *ACAT*2, and *ACAT*1 of WT, OE::*gat*1, and $\Delta gat1$ in the B80:3 group. *PDC*, encoding pyruvate decarboxylase; *ACS*, encoding acetyl-CoA synthetase; *ICL*1 and *ICL*2, encoding isocitrate lyase 1 and 2; *ACAT*1, encoding acetyl-CoA acyltransferase 1; *ACAT*2, encoding acetyl-CoA acyltransferase 2. **c** Yeast one-hybrid assays to detect whether SpGAT1 bound to promoters of *PDC*, *ACS*, *ICL*1, *ICL*2, *ACAT*2, and *ACAT*1. Y1H Gold [pAbAi-gene promoter + pGADT7] is the negative control, and Y1H Gold [pAbAi-gene promoter + pGADT7-SpGAT1] is the experiment group. Data are presented as the means ( SE, $n = 3$). ns: no significant difference, *$p < 0.01$, **$p < 0.001$, ***$p < 0.0001$

We found SpGAT1 had distinct regulation patterns on these genes under different C/N ratio mediums (Fig. 4). SpGAT1 positively regulated *ACS* in the pyruvate bypass pathway and *ICL*1/*ICL*2 in the glyoxylate shunt pathway, irrespective of the nitrogen concentration. Besides, SpGAT1 could positively regulate *ACAT*2 expression only in a low C/N ratio medium (Fig. 4). In particular, yeast one-hybrid assays showed that SpGAT1 directly bound to promoters of *ACAT*2, *PDC*, *ACS*, *ICL*1, and *ICL*2, except *ACAT*1 (Fig. 4c). In the low C/N ratio medium, transcription levels of *ICL*1, *ICL*2, *ACAT*1, *ACS*, and *ACAT*2 of OE::*gat*1 increased (approximately 1.8, 3.1, 3.9, 1.8, and 2.7 times, respectively); while transcription levels of *ICL*2, *ACAT*1, *ACS*, and *ACAT*2 of $\Delta gat1$ reduced 0.4-, 0.4-, 0.5-, and 0.2-fold versus WT, respectively. In the high C/N ratio medium, *ICL*1, *ICL*2, and *ACS* expression levels in OE::*gat*1 were significantly up-regulated 21-, 2.8-, and 4-fold, respectively, but expression levels of *ICL*1, *ICL*2, and *ACAT*2 of $\Delta gat1$ did not change significantly; *ACS* and *ACAT*1 expression levels both down-regulated about 0.5-fold. In the OE::*gat*1 and $\Delta gat1$ strains, *PDC* exhibited a special transcription pattern and its transcription level dropped 0.4- and 0.3-fold in the low C/N ratio medium and up-regulated by 8- and 83-fold in the high C/N ratio medium, respectively (Fig. 4a,b). Therefore, it was unclear how SpGAT1 regulated *PDC*.

### SpGAT1 interacts with SpMIG1 to affect carbon source utilization rate to promote lipid metabolism

There was no overall substantial decrease in the expression levels of lipid biosynthesis key genes on the high C/N ratio medium (Figs. 3f and 4b), but a significant decrease in sugar consumption rate and biomass resulted in a major loss in lipid yield in the $\Delta gat1$ strain compared to WT (Fig. 3b). These findings demonstrated that transcripts of genes involved in lipid biosynthesis did not always correlate with lipid accumulation, suggesting that additional factors other than SpGAT1’s direct activation might be engaged in SpGAT1’s influence on lipid biosynthesis.

**Fig. 5:**
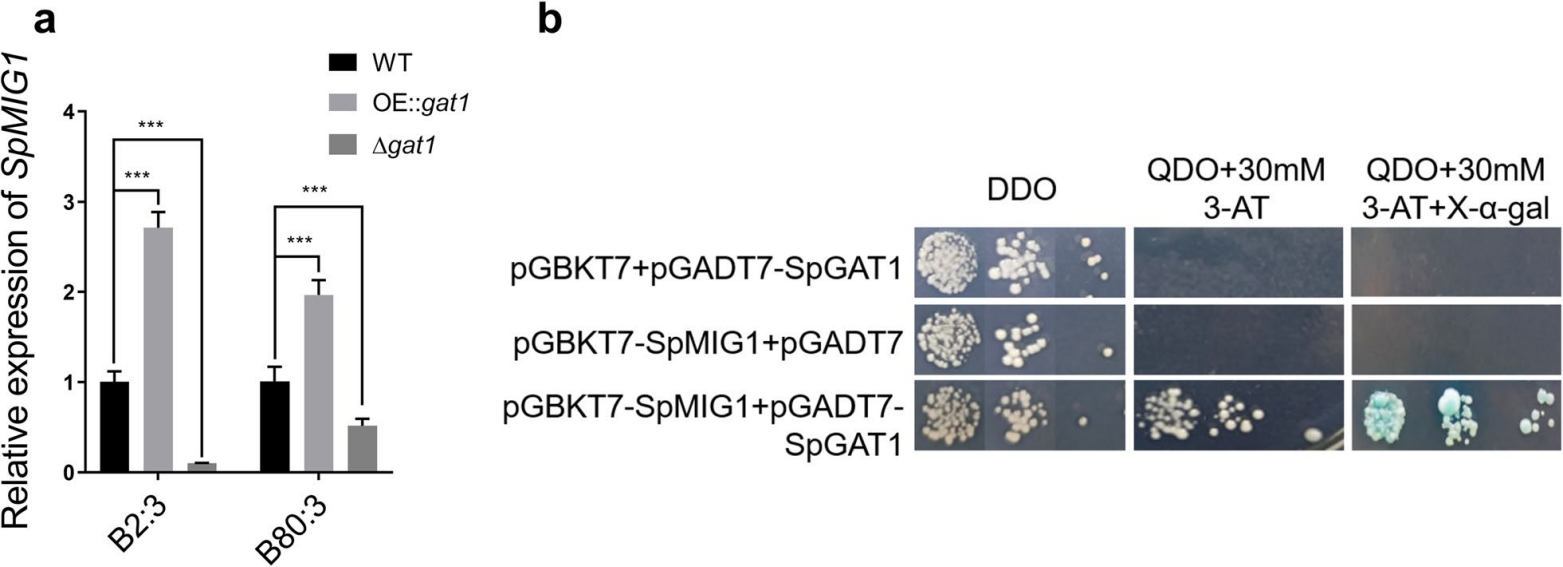
SpGAT1 interacted with SpMIG1 and these two genes had a positive regulation relationship. **a** Expression profiles of *SpMIG*1 in WT, OE::*gat*1, and $\Delta gat1$ in the B2:3 and B80:3 groups. **b** Yeast two-hybrid assays to detect whether SpGAT1 had an interaction with SpMIG1. Y2H Gold [pGBKT7+ pGADT7-SpGAT1] and Y2H Gold [pGBKT7-SpMIG1+pGADT7] are negative control, Y2H Gold [pGBKT7-SpMIG1+pGADT7-SpGAT1] is experiment group. DDO: SD/-Leu/-Trp, QDO: SD/-Leu/-Trp/-His/-Ade, 3-AT: 3-amino-1,2,4-triazole. Data are presented as the means ( SE, $n = 3$). ns: no significant difference, *$p < 0.01$, **$p < 0.001$, ***$p < 0.0001$

We questioned if SpGAT1 impacted the expression of genes involved in carbon uptake. Using $S.\ cerevisiae$ as reference species, we predicted possible interactions between GAT1 and MIG1 in the String database (Fig. S5). Yeast two-hybrid assays revealed that SpGAT1 interacted with SpMIG1 (Fig. 5b). To learn more about how SpGAT1 regulates carbon metabolism, we examined *SpMIG*1 expression in the WT, $\Delta gat1$, and OE::*gat*1 strains in different C/N ratio mediums. Compared to WT, *SpMIG*1 expression in OE::*gat*1 was up-regulated to 2.7- and 2.0-fold, and *SpMIG*1 expression of $\Delta gat1$ was down-regulated to 0.1- and 0.5-fold in the B2:3 and B80:3 groups, respectively (Fig. 5a). To conclude, knockout *SpGAT*1 significantly reduced lipid accumulation by negatively regulating the expression of *SpMIG*1, which might impair glucose consumption rate in $S.\ podzolica$ zwy-2- 3.

## Discussion

**Fig. 6:**
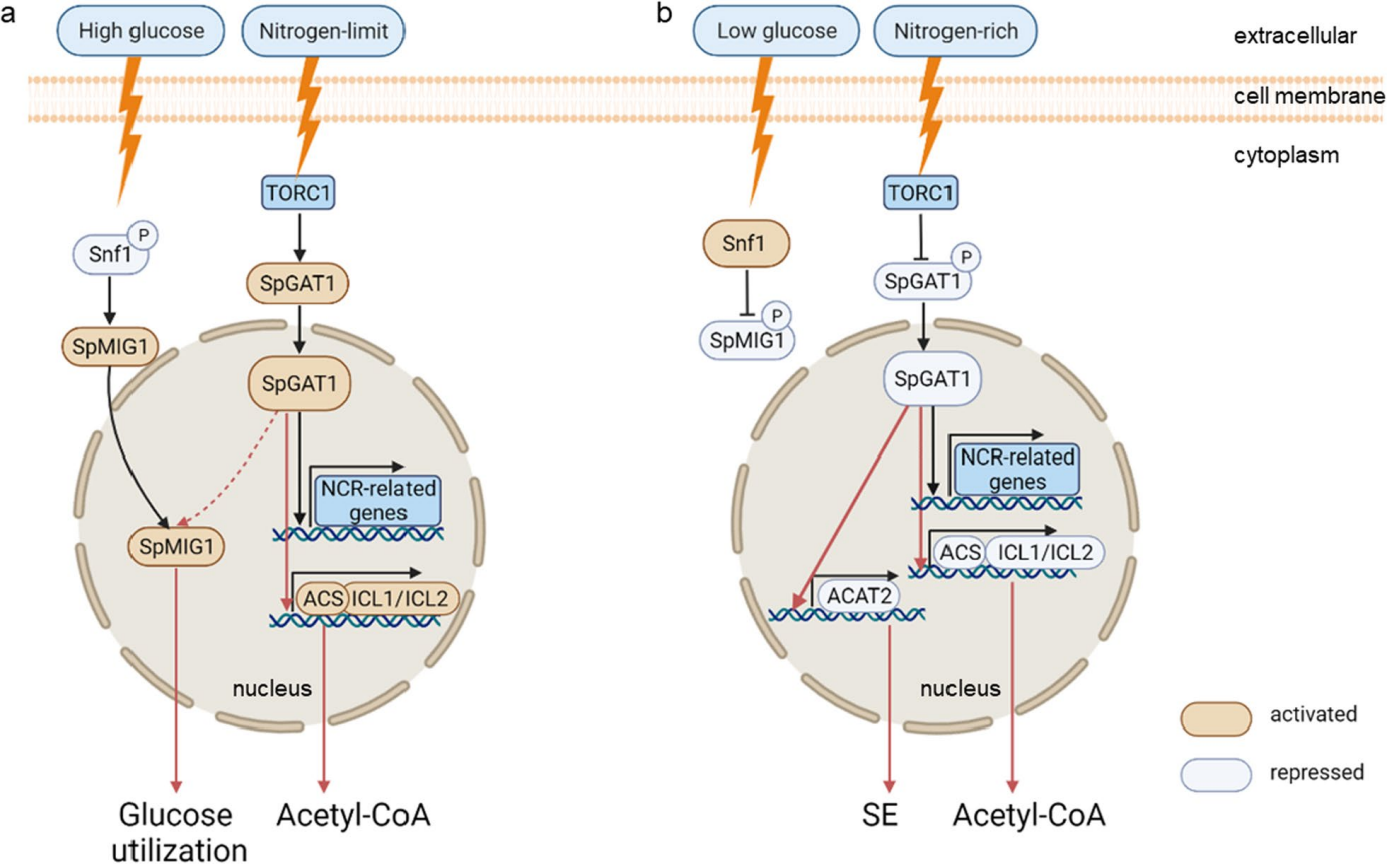
Hypothetic SpGAT1 regulation pattern diagrams in $S.\ podzolica$ zwy-2-3 lipid metabolism under high and low C/N ratio conditions. **a** High C/N ratio condition, **b** low C/N ratio condition. TORC1: target of rapamycin complex 1; NCR: nitrogen catabolite repression; SpGAT1: GATA-1-type zinc finger DNA-binding motif protein; SpMIG1: $\mathrm {C_2H_2}$ zinc fingers transcriptional factor; SNF1: sucrose non-fermenting related kinase 1; *ACS*: encoding acetyl-CoA synthetase; *ICL*1 and *ICL*2, encoding isocitrate lyase 1 and 2; *ACAT*2, encoding acetyl-CoA acyltransferase 2; SE: sterol esters. Red arrows represent hypothetic positive regulations proposed in this paper, black arrows represent the known regulatory pathways, and the dotted line in Fig. 6a represents the uncertain upstream and downstream relationship between SpGAT1 and SpMIG1

In this study, the effects of SpGAT1, a GATA-type transcriptional activator, on carbon absorption and lipid biosynthesis in $S.\ podzolica$ zwy-2-3 were investigated. As shown in Fig. 6, SpGAT1 could regulate carbon metabolism by interacting with SpMIG1; then, SpGAT1 could bind to promoters of key genes in the pyruvate bypass and glyoxylate shunt pathways to regulate cytoplasmic acetyl-CoA levels, and finally, SpGAT1 had different regulation patterns on $S.\ podzolica$ zwy-2-3 carbon and lipid metabolism in low and high C/N ratio conditions.

SpGAT1 was found to regulate $S.\ podzolica$ zwy-2-3 carbon source uptake and utilization to promote lipid synthesis, potentially by interacting with SpMIG1, a CCR transcription repressor(Fig. 6a). The $\Delta gat1$ strain had significantly lower biomass and glucose consumption ability, as well as worse growth on mannose, sucrose, xylose, and xylan carbon sources than WT and OE::*gat*1 (Fig. 3b,d and Additional file 2: Fig. S3c). In $Fusarium \ graminearum$, $Pleurotus \ ostreatus$, and $Aureobasidium \ pullulans$, knockout of either *GAT*1 or *AreA* also resulted in a profound decrease in growth rate [31–33]. Previous research has reported that SNF1-MIG1 pathway is a crucial regulatory pathway for glucose utilization, and deletion of *MIG*1 might hinder glucose metabolism capacity, resulting in a prolonged lag period [28, 34], while carbon absorption rate is strongly related to lipid synthesis [35]. Our results found that SpGAT1 interacted with SpMIG1 and deletion of *SpGAT*1 drastically lowered *SpMIG*1 expression (Fig. 5). Hence we hypothesized that SpGAT1 could regulate sugar utilization capacity and lipid accumulation by interacting with SpMIG1. Under low glucose concentrations, MIG1 is phosphorylated by activated SNF1 and localizes to the cytoplasm; conversely, under high glucose concentrations, SNF1 kinase is inhibited, leading MIG1 to be dephosphorylated and relocated to the nucleus, inhibiting downstream gene expression [36]. Reduced expression level of *SpMIG*1 caused by knockout *SpGAT*1 had an extremely pronounced effect on glucose utilization related genes in the high C/N ratio (high glucose concentration) medium, eventually leading to a decrease in total biomass and lipid yield (Fig. 3a, b); however, this phenomenon did not appear in the low C/N ratio (low glucose concentration) medium, probably because SpMIG1 was localized in the cytoplasm by SNF1 phosphorylation under low C/N ratio conditions. Actually, upstream regulatory genes (TOR1, SIT4, URE2) of GAT1 in the TOR signaling pathway regulated yeast lipid metabolism [21]. TORC1 and carbon source uptake regulator SNF1 (an upstream regulator of MIG1) had a close regulatory interaction [37, 38]. Higher TORC1 activity was frequently linked with lower SNF1 activity, vice versa [23]. During carbon deprivation, SNF1 also regulated the hyperphosphorylation of GAT1 and GLN3 to transfer their sublocations [39]. Furthermore, mutations of GATA-type transcription factors in $Y.\ lipolytica$ disrupted lipid accumulation which could be regulated by MIG1 to suppress CCR-related gene expression [40]. Several studies implied that genes of the TOR signaling pathway could regulate carbon metabolism through the SNF1-MIG1 signaling pathway. Therefore, our result of SpGAT1 and SpMIG1 interaction further revealed a tight link between nitrogen source, carbon source, and yeast lipid metabolism.

SpGAT1 had different regulation patterns for lipid metabolism in low and high C/N ratio conditions. Carbon and nitrogen are the two most abundant nutrient elements for all living organisms, and their metabolism is tightly coupled. Properly balanced metabolism of carbon and nitrogen (like B2:3 medium) was necessary for optimal growth, while under the nitrogen-limit condition (like B80:3 medium), mass-accumulated $\alpha$-ketoglutaric acid, as a carbon skeleton in nitrogen assimilation, entered TCA cycle to mediate the homeostasis of carbon and nitrogen metabolism and relieved restricted growth caused by nitrogen limitation [41]. The related metabolic pathways were ultimately coordinated by the master transcription factors that sense the intracellular metabolites [42]. GAT1, as a key transcription factor promoting the uptake and utilization of nitrogen sources, could play an important function in the transcriptional regulation of carbon and nitrogen metabolism. Our findings suggested that *SpGAT*1 transcription level was substantially positively related to lipid synthesis genes expression levels, and lipid accumulation in both nitrogen limitation and rapamycin treatment indicated that SpGAT1 was likely to regulate lipid metabolism by NCR and TOR pathway (Fig. 2). But although, there were diverse regulation patterns, in a nitrogen-rich medium, overexpression of the *SpGAT*1 promoted lipid formation (Fig. 3b), which may raise the nuclear-located SpGAT1 levels and stimulate transcriptional activation of downstream genes. In particular, under nitrogen-rich conditions, lipid biosynthesis genes in the OE::*gat*1 strain were typically up-regulated as *SpGAT*1 expression levels increased. In contrast, same genes in the $\Delta gat1$ strain were generally down-regulated (Fig. 3e). As a result, the lipid yield of the OE::*gat*1 strain raised from 10 % to 30 % compared to WT (Fig. 3c). Under nitrogen-limited conditions, deletion of *SpGAT*1 affected nuclear localization of SpGAT1, which reduced transcriptional activation of downstream genes and lipid accumulation (Fig. 3b); however, only *FAS*1 and *ACC* expression were severely down-regulated in the $\Delta gat1$ strain (Fig. 3f), most likely owing to the compensatory effect of other nitrogen source response factors such as GLN3; the decrease in lipid yield of $\Delta gat1$ strain mostly due to a loss in biomass (Fig. 3a–c). Besides, because of *SpGAT*1’s high background expression level in the OE::*gat*1 strain, lipid synthesis genes were marginally up-regulated and lipid yield did not alter significantly in the high C/N ratio medium (Fig. 3b,f). Overall, SpGAT1 could only up-regulate the expression of several lipid metabolism genes when the nitrogen source was sufficient, which had no effect on organism growth; while in the case of nitrogen limitation, SpGAT1 not only promoted the expression of genes related to the uptake and utilization of nitrogen sources but also could regulate the assimilation of carbon sources by interacting with SpMIG1 to alleviate growth inhibition.

SpGAT1 bound to promoters of *ACS* in the pyruvate bypass pathway, *ICL*1/*ICL*2 in the glyoxylate shunt pathway, and *ACAT*2 in different patterns that were also dependent on the C/N ratios to modulate cytoplasmic acetyl-CoA levels. Among those genes, SpGAT1 positively regulated *ICL*1, *ICL*2, and *ACS* to increase lipid accumulation, irrespective of nitrogen concentrations (Fig. 4a,b). Increasing cytoplasmic acetyl-CoA levels, a crucial precursor of TAG production, is a smart way to boost lipid yield. Overexpression of *ACS* in $Chromochloris \ zofingiensis$, $C.\ reinhardtii$ and *Schizochytrium* sp. TIO1101 greatly increased the intracellular acetyl-CoA levels [43–45]. ICL is an enzyme of fatty acid metabolism, such as in oleaginous seed plants, the glyoxylate cycle was essential during seed germination for conversion of fatty acids into sugars via $\beta$-oxidation of fatty acids and gluconeogenesis [46]; ICL1 and 2 were key enzymes for $Mycobacterium\ tuberculosis$ growth and virulence in vivo [47]. Therefore, deletion of *ICL* increased the lipid content in many microorganisms [46, 47]. However, research also found that reductions in the levels of TCA cycle intermediates when *ICL* was deficient in $M.\ tuberculosis$ [48]; in $Y.\ lipolytica$, overexpression of *ICL*1 increased the ratio of citric acid production [49]. In oleaginous yeast, the more lipid accumulated, the more lipid degraded, relatively; and the consumption of fatty acids required the regulation of the carbon flux bifurcation between the TCA cycle and the glyoxylate shunt. In this context, the glyoxylate shunt was significantly up-regulated to maintain the TCA cycle [46, 50]. Therefore, SpGAT1 may positively regulate *ICL*1 and *ICL*2 to speed up the TCA cycle and accelerated citric acid storage in the cytoplasm, which could return to raise cytoplasmic acetyl-CoA levels in $S.\ podzolica$ zwy-2-3 [51–53]. Furthermore, SpGAT1 may influence the expression of *ACAT*2, which encoded a key enzyme for HMG-CoA production, to promote sterol ester (SE) synthesis only when the C/N ratio was low (Fig. 4a, c). Other research discovered that knocking out *ACAT*2 impeded the development of sheep precursor adipocytes, and knockout cells had less intracellular lipid droplets than *ACAT*2 overexpression cells and WT cells [54]. *RKACAT*2 overexpression increased carotenoid synthesis in $Rhodosporidium\ kratochvilovae$. Expressing a truncated *HMGR* and *ERG*10 (homologous gene of *ACAT*2) in $S.\ cerevisiae$ enhanced the mevalonate pathway and $\alpha$-bisabolol titer by 2.9-fold [55]. The expression levels of *ACAT*1, *ACAT*2, *ICL*1, *ICL*2, *ACS*, and lipid synthesis-related genes in the $\Delta gat1$ strain were too low in the low C/N ratio medium (Figs. 3e and 4a), implying that the acetyl-CoA level used for lipid synthesis was already extremely low, and there was no more acetyl-CoA to consume to maintain $S.\ podzolica$ zwy-2-3 metabolic homeostasis. In contrast, the levels of intracellular acetyl-CoA increased in the OE::*gat*1 strain because of increased expressions of *ACAT*2, *ACAT*2, *ICL*1, *ICL*2, *ACS*, and genes associated with lipid synthesis (Figs. 3e and 4a). Lipid accumulation requires a constant supply of carbon sources [56, 57], but the carbon source in the medium was virtually depleted at the late fermentation stage. Therefore, ACAT1 could promote fatty acid degradation and increased levels of $\beta$-oxidation, resulting in the release of acetyl-CoA. Finally, with positively regulating *ACAT*2, SpGAT1 returned acetyl-CoA to SE synthesis. However, the expression of *ACAT*2 did not change significantly in the high C/N ratio media (Fig. 4b), most likely due to the preponderance of TAG accumulation in the nitrogen-limit medium.

Under differing C/N ratios in combination with rapamycin treatment, SpGAT1 had no influence on the fatty acid profiles and had less effect on fatty acid unsaturation in $S.\ podzolica$ zwy-2-3 (Additional file 2: Figs. S1b and S3a). When $T.\ oleaginosus$ was grown on the YPD medium, the unsaturated fatty acid of the rapamycin-treated group rose about 15 % compared to the untreated group, but fell about 30 % compared to the nitrogen-limited culture group [20]. When $S.\ podzolica$ zwy-2-3 was grown on the B2:3 medium, the ratio of C18:1 and C18:2 fatty acids increased by less than 5 % following rapamycin treatment, and its unsaturation was substantially higher than that in the B80:3 group. Moreover, the unsaturated fatty acid ratios between OE::*gat*1, $\Delta gat1$, and WT varied little. In conclusion, the fatty acid profiles of $S.\ podzolica$ zwy-2-3 following diverse treatments practically maintained invariance, and this characteristic was extremely suited for biodiesel production.

In addition, SpGAT1 had a relationship with different carbon and nitrogen sources utilization. On non-preferred nitrogen sources, the $\Delta gat1$ strain grew worse than WT and OE::*gat*1 strains (Additional file 2: Fig. S3b). In $Candida\ albicans$, knockout *GLN*3 grew poorly on proline [58], while in $Aspergillus\ nidulans$, AreA (homologous gene of GAT1) nuclear localization signal sequence mutation dramatically influenced its growth under various nitrogen sources [59], and deletion of *GAT*1 in $A.\ pullulans$ also made its growth significantly worse on alanine, phenylalanine, leucine, isoleucine, threonine, tryptophan, and other amino acids, however, there was no significant difference in growth on inorganic nitrogen and preferred amino acids [33]. Therefore, it was probable that the deleting *SpGAT*1 weakened the metabolic capacity to regulate nitrogen sources, making microorganisms impossible to properly utilize non-preferential nitrogen sources. *AreA* knockdown significantly reduced the growth rate of $F.\ graminearum$ on glutamic acid, glutamine, and inorganic nitrogen [31]; knockout *GAT*1 in $A.\ pullulans$ also decreased biomass by 36.6 % in a medium with C/N = 90:2, but biomass decrease steadily declined as the nitrogen source concentration increase [33]. In the high C/N ratio medium, $\Delta gat1$ strain also showed poorer utilization of fermentable carbon sources (glucose, sucrose, and mannose) and non-fermentable carbon sources (xylose and xylan) than WT and OE::*gat*1 strains (Additional file 2: Fig. S3c). In $P.\ ostreatus$, knockout *GAT*1 either led to faulty zygotic development, sluggish growth rate, and drastically impaired lignin degradation capacity [32]. We speculated that SpGAT1 regulated kinds of sugar utilization capacity through interacting with CCR-related transcriptional factor SpMIG1. However, why there was a similar trend between fermentable and non-fermentable carbon sources and whether the overexpression strain OE::*gat*1 had a stronger oil production capacity than WT in xylan needed to be further investigated.

SpGAT1 enhanced lipid accumulation under high C/N ratio conditions by interacting with SpMIG1 and positively regulating *ICL*1, *ICL*2, and *ACS* (Fig. 6a). Under low C/N ratio conditions, SpGAT1 increased lipid yield by positively promoting expression levels of *ACAT*2, *ICL*1, *ICL*2 and *ACS* (Fig. 6b). This paper is the first to study how the transcription factor SpGAT1 regulated lipid metabolism and the interaction between SpGAT1 and SpMIG1, which might serve as a catalyst for much more research into lipid metabolism regulated by nitrogen and carbon metabolism cross-talk.

## Conclusion

In conclusion, SpGAT1, a vital transcription factor of the NCR and TOR signaling pathway, could regulate lipid and carbon metabolism in $S.\ podzolica$ zwy-2-3. On the one hand, SpGAT1 displayed divergent lipid metabolism regulatory patterns. *SpGAT*1 deletion reduced lipid yield by about 50 % in the high C/N ratio medium, but *SpGAT*1 overexpression boosted lipid yield by around 200 % in the low C/N ratio medium. On the other hand, the transcription factor SpGAT1 regulated carbon uptake and utilization to promote lipid formation via interacting with SpMIG1, an important transcription factor of carbon metabolism. SpGAT1 also increased cytoplasmic acetyl-CoA level to promote lipid accumulation through positively regulating *ACS* and *ICL*1/*ICL*2 in the pyruvate bypass and glyoxylate shunt pathways, respectively. However, further research is necessary to confirm how SpGAT1 influences the utilization of carbon sources via SpMIG1. Overall, these findings add to our knowledge of non-conventional oleaginous yeast lipid metabolism.

## Methods

### Preparation of strains and inoculum

All experiments were performed using the yeast strain $S.\ podzolica$ zwy-2-3 SCTCC300292 obtained from the Sichuan Province Typical Culture Collection center. YPD medium (called B2:3 medium in this paper, glucose 20 g/L, yeast extract 10 g/L, peptone 20 g/L) and nitrogen-limit medium (called B80:3 medium in this paper, glucose 80 g/L, yeast extract 3 g/L, $\mathrm {MgSO_4\cdot 7H_2O}$ 1.5 g/L) were used for cultivation. 25 mg/mL rapamycin (Coolaber, BC grade) was dissolved in DMSO.

For pre-culture, seed cells were inoculated in YPD medium for 2 days at 28 $^{\circ }$C, 180 rpm, then re-inoculated at a 1 % ratio into 50 mL YPD medium and cultured for 24 h. For fermentation, seed cells were inoculated in YPD medium and nitrogen-limit medium at a ratio of 2 % and cultured for 5 days in 500-mL baffled shake flasks. All the equipment was steam-sterilized at 121 $^{\circ }$C for 20 min, and the medium was steam-sterilized at 115 $^{\circ }$C for 15 min.

### Determination of biomass and lipid

Cells were collected after fermentation by centrifugation (12000 rpm, 5 min) and dried to constant weight. Cellular total lipid was extracted by chloroform and methanol with the modified Folch method [12]. Drying cell pellets were pulverized and shaken for 12 h with 15 mL 4 mol/L ${\mathrm{HCl}}$. Then, the mixture was then treated three times for 10 min each in an ice bath and a boiling water bath. Subsequently, cell lysate was mixed with 10 mL Folch reagent (2:1 chloroform/methanol) in a 50-mL tube and treated for 6 h. Finally, after centrifuging at 8000 rpm for 6 min, transferred chloroform layer into a clean tube and dried for 2–3 days.

### Analysis of fatty acid composition by GC–MS

Methyl esterification of fatty acid: extracted yeast lipid(1 ul) was mixed with 2 mL hexane and 2 mL 0.6 mol/L $\mathrm {KOH}$-$\mathrm {CH_3OH}$ in a tube. Then, vortexed samples for 2 min and incubated for 15 min. Next, added 5 mL dd$\mathrm {H_2O}$ to allow the layers to separate, then hexane layers were transferred into a clean tube. GC–MS analysis: fatty acid methyl esters were analyzed on GC-MS-QP2010 and HP-88.

### RNA extract and qRT-PCR gene expression

RNA extracted with TRIzol method. RNA reverse transcription method was according to Takara Primer ScriptTM RT reagent Kit with gDNA Eraser Reverse Transcription Kit instructions. The real-time RT-PCR method was according to Takara TB Green$^{TM}$ Premix Ex Taq$^{TM}$ II instructions and the CFX96 Real-Time PCR Detection System User Guide.

### Identification, cloning, and bioinformatics analysis of SpGAT1

SpGAT1 was identified from the $S.\ podzolica$ reference genome (DSM27192) because $S.\ podzolica$ zwy-2-3 was earlier isolated from our laboratory without genomic data, its RNA-seq data have not annotated and spliced the full length of *SpGAT*1. Then, RSH87488.1 in the DSM27192 strain was identified as SpGAT1. Using the CDS sequence of gene which encoded RSH87488.1 protein as a reference, primers were designed to clone the full-length SpGAT1 in $S.\ podzolica$ zwy-2-3. SpGAT1 sequence was analyzed by bioinformatics after amplification, sequencing, and blast with RSH87488.1. The open reading frame of *SpGAT*1 was predicted using ORF-Finder, the conserved structural domain was predicted using Pfam, and the isoelectric point and molecular weight of SpGAT1 were analyzed using the Expasy.

### Construction of $\Delta gat1$ and OE::*gat*1 strains

To knockout *SpGAT*1, a 3373 bp fragment of *SpGAT*1 extending from positions +270 bp to +3643 bp was replaced with hygromycin resistance cassette HYG based on a homologous recombination method. First, we generated the *SpGAT*1 knockout cassette by overlapping the upstream and downstream homologous arms as well as the hygromycin resistance gene; then, ligating the *SpGAT*1 knockout cassette to the pMD19T vector to construct the knockout vector KO-SpGAT1. After that, KO-SpGAT1 was transformed into $S.\ podzolica$ zwy-2-3 competent cells by electroporation. To construct the *SpGAT*1 overexpression strain, we cloned the $S.\ podzolica$ zwy-2-3 own glyceraldehyde-3-phosphate dehydrogenase promoter and combined the promoter, the tCYC1 terminator, and the full-length of the *SpGAT*1 gene by overlapping PCR to construct the *SpGAT*1 overexpression cassette. Inserting the *SpGAT*1 overexpression cassette between the *Spe*I and *Hind*III sites of the prf-HU2 plasmid and transforming DH5$\alpha$ after recombinase ligation, then the successfully generated overexpression vector prf-HU2-OE-SpGAT1 transformed into the $S.\ podzolica$ zwy-2-3 through Agrobacterium tumefaciens Mediated Transformation (ATMT). Both OE::*gat*1 and $\Delta gat1$ strains were screened on YPD with 100 $\mu$g/mL hygromycin. Verified right *SpGAT*1 knockout and overexpression strains with genomic PCR, the knockout and overexpression transformants were purified and rescreened on YPD (200 $\mu$g/mL hygromycin) medium.

### Detection utilization abilities of different carbon and nitrogen sources

Cell suspensions of constructed mutant strains OE::*gat*1 and $\Delta gat1$ as well as WT strains were diluted at 0.1, 0.01, and 0.001 for serial dilution spot tests. For carbon utilization tests, cell suspensions were spotted on a B80:3 medium containing different carbon sources (glucose, sucrose, mannose, xylose, xylan) for 2 d; To test utilization of different nitrogen sources, cell suspensions were spotted on 10 % glucose YCB (yeast carbon base) medium containing different amino acids and incubated for 3 days with 2 mM of each amino acid, 20 mM urea and 37 mM $\mathrm {(NH_4)_2SO_4}$.

### Subcellular localization of SpGAT1 and SpMIG1

To construct subcellular localization vectors, the full length of the *SpGAT*1 and *SpMIG*1 were amplified using the primers in Additional file 1: Table S2 and inserted between the pYES2-GFP plasmid *Kpn*I and the *Bam*HI digestion sites. Then constructed pYES2-SpGAT1-GFP and pYES2-SpMIG1-GFP vectors were transformed into BY4741 ($MAT\alpha \ his3 \Delta 1\ leu2\ met15 \Delta \ ura3-52$) using the PEG/LiAc transformation method and spread on SD/-Ura plate to screen positive transformants.

To study the subcellular localization of transcription factor SpGAT1 and SpMIG1, BY4741-pYES2-SpGAT1 and BY4741-pYES2-SpMIG1-GFP were inoculated into SD/-Ura containing 2 % glucose until OD600 = 1.2-1.4 at 30 $^{\circ }$C, 180 rpm; collecting cell pellet with 1000 rpm, for 5 min and incubating cells with SD/-Ura without sugar until intracellular glucose has been exhausted; then transferring cells to SD/-Ura containing 2 % galactose and incubated for 5 h. Subcellular localization of SpGAT1 and SpMIG1 were detected under fluorescence microscopy after DAPI staining.

### TFBS Tools predicts lipid metabolism genes regulated by transcription factor SpGAT1

Installed the R packages TFBS Tools, JASPAR2018, and Biostrings firstly, then used TBtools to extract gene promoter sequences from the reference genome DSM27192. The promoter sequences of genes with high scores (set score = ”90 % ” , strand = ” +” ) were then screened as candidate genes; lastly, key lipid metabolism genes were rescreened from these candidate genes using the dynamic GO enrichment analysis tool on omicShare website.

### Yeast one-hybrid and yeast two-hybrid assays

For yeast two-hybrid assays, refer to the Matchmaker$\circledR$ Gold Yeast Two-Hybrid System User Manual for operating instructions. First, *SpGAT*1 and *SpMIG*1 were cloned using the primers in Additional file 1: Table S2; then, *SpGAT*1 was inserted into the pGADT7 vector between the *EcoR*I and *BamH*I digestion sites and *SpMIG*1 was inserted into the pGBKT7 vector between the *Nde*I and *BamH*I digestion sites to form the bait and prey constructs. The negative control (pGADT7-SpGAT1 and pGBKT7) and (pGADT7 and pGBKT7-SpMIG1) and the experimental group (pGADT7-SpGAT1 and pGBKT7-SpMIG1) were co-transformed into Y2H with PEG/LiAc method according to the manufacturer’s instructions (Clontech), and spread on SD/-Leu/-Trp to screen positive transformants; Finally, Y2H [pGADT7-SpGAT1+pGBKT7], Y2H [pGADT7 +pGBKT7-SpMIG1] and Y2H [pGADT7-SpGAT1+pGBKT7-SpMIG1] were diluted at 0.1, 0.01, and 0.001 for serial dilution and spotted on DDO, TDO, QDO, and QDO+30mM 3-AT plates to detect the interaction of SpGAT1 with SpMIG1, respectively. 3-AT was for suppressing self-activation of prey protein.

For yeast one-hybrid assays, refer to the Matchmaker$\circledR$ Gold Yeast one-hybrid Library Screening System User Manual instructions. pGADT7-GAT1 vector has been constructed in yeast two-hybrid assays, the promoter sequences of *PDC*, *ICL*1, *ICL*2, *ACAT*1, *ACS* and *ACAT*2 were inserted into the pAbAi vector. Then, linearize pAbAi-promoter vectors with BstB*I* digestion sites and transform linearized vectors into Y1H, the positive transformants were gradiently diluted at 0.1, 0.01, and 0.001 for serial dilution and spotted on SD/-Ura and SD/-Ura/AbA to detect promoters’ self-activation. Next, the negative control (pGADT7) and the experimental group (pGADT7-SpGAT1) were transformed into Y1H [pAbAi-promoter] competent cells according to the manufacturer’s instructions (Clontech); finally, the positive transformants were gradiently diluted at 0.1, 0.01, and 0.001 for serial dilution and spotted on SD/-Leu and SD/-Leu/AbA to detect whether SpGAT1 bound to the promoter.

## Supplementary Information

**Additional file 1: Table S1.** Strains and plasmids. **Table S2.** Primers for gene amplification and qRT-PCR.** Table S3.** cDNA sequence of SpGAT1 and SpMIG1.

**Additional file 2: Fig. S1.** Single cell lipid yield and fatty acid profile of S.podzolica zwy-2-3 cultivated in rapamycin treatment and different C/N ratio mediums. a Single cell lipid yield stained by Nile red. b Fatty acid profiles analyzed by GC-MS.** Fig. S2.** Results of SpGAT1 mutant identification. a Results of amplifying hygromycin gene, homologous arm, and part of SpGAT1 gene (lane 1: marker 5000 bp, lane 2: amplifying hygromycin gene and LB homologous arm in WT, lane 3: amplifying hygromycin gene and LB homologous arm in Δgat1, lane 4: amplifying hygromycin gene and RB homologous arm in WT, lane 5: amplifying hygromycin gene plus RB homologous arm in Δgat1, lane 6: amplifying part of SpGAT1 gene in Δgat1, lane 7: amplifying part of SpGAT1 gene in WT). b Results of amplifying full length of SpGAT1 (lane 1: marker 2000 bp, lane 2: WT, lane 3: Δgat1). c Results of amplifying hygromycin gene by colony PCR (lane 1: marker 5000 bp, lane2: OE::gat1, lane 3: WT). d Detecting SpGAT1 expression level in WT and OE::gat1 by qRT-PCR.** Fig. S3.** Effects of SpGAT1 on fatty acid profiles and different carbon/nitrogen sources utilization in S.podzolica zwy-2-3. a Fatty acid profiles in WT, Δgat1 and OE::gat1 cultivated in low and high C/N ratio mediums. b Utilization different nitrogen sources in WT, Δgat1, and OE::gat1. c Utilization different carbon sources in WT, Δgat1, and OE::gat1.** Fig. S4.** Results of subcellular localization observed by fluorescence microscopy. Cyto: transcription factors were cytoplasmic localization, Nuclear: transcription factors were nuclear localization, Nucl-Cyto: transcription factors were located in cytoplasm and nuclear.** Fig. S5.** Prediction of SpGAT1 interacting protein. a Predicted by GeneMANIA database, b Predicted by String database.

## Data Availability

All data generated or analyzed during this study are included in this published article and its additional files.

## Abbreviations

GAT1: GATA-1-type zinc finger DNA-binding motif protein, homologous protein of AreA
MIG1: $\mathrm {C_2H_2}$ zinc fingers transcriptional factor, homologous protein of CreA
SNF1: Sucrose non-fermenting related kinase 1
TOR: Target of rapamycin
FAS1: Fatty acid synthase
ZWF1: Glucose-6-phosphate dehydrogenase
G3PDH: Glycerol-3-phosphate dehydrogenase
ACS: Acetyl-CoA synthetase
ACC: Acetyl-CoA carboxylase
SIT4: Serine/threonine-protein phosphatase
ACAT1: Acetyl-CoA acetyltransferase 1
ACAT2: Acetyl-CoA acetyltransferase 2
ICL1: Isocitrate lyase 1
ICL1: Isocitrate lyase 1
PDC: Pyruvate decarboxylase
TAG: Triacylglycerol
SE: Sterol esters
NCR: Nitrogen catabolite repression
CCR: Carbon catabolite repression

## References

[1] Brandenburg J, Blomqvist J, Shapaval V, Kohler A, Passoth V (2021). Oleaginous yeasts respond differently to carbon sources present in lignocellulose hydrolysate. Biotechnol Biofuels..

[2] Liang MH, Jiang JG (2013). Advancing oleaginous microorganisms to produce lipid via metabolic engineering technology. Prog Lipid Res.

[3] Sreeharsha RV, Mohan SV (2020). Obscure yet promising oleaginous yeasts for fuel and chemical production. Trends Biotechnol.

[4] Wang YA, Zhang SF, Zhu ZW, Shen HW, Lin XP, Jin X, Jiao X, Zhao ZK (2018). Systems analysis of phosphate-limitation-induced lipid accumulation by the oleaginous yeast rhodosporidium toruloides. Biotechnol Biofuels.

[5] Spagnuolo M, Yaguchi A, Blenner M (2019). Oleaginous yeast for biofuel and oleochemical production. Curr Opin Biotechnol.

[6] Xu H, Zhao N, Yao H, Qin H, Zeng J, Ran Y, Yang Y, Qiao D, Cao Y (2019). Lipid production from corn stover by a cost-efficient system featuring ammonium carbonate-steam explosion and recirculating enzymatic hydrolysis. Biomass Bioenergy.

[7] Qian X, Gorte O, Chen L, Zhang W, Dong W, Ma J, Jiang M, Xin F, Ochsenreither K (2019). Co-production of single cell oil and gluconic acid using oleaginous cryptococcus podzolicus dsm 27192. Biotechnol Biofuels.

[8] Aliyu H, Gorte O, Neumann A, Ochsenreither K (2021). Global transcriptome profile of the oleaginous yeast saitozyma podzolica dsm 27192 cultivated in glucose and xylose. J Fungi..

[9] Gorte O, Kugel M, Ochsenreither K (2020). Optimization of carbon source efficiency for lipid production with the oleaginous yeast saitozyma podzolica dsm 27192 applying automated continuous feeding. Biotechnol Biofuels.

[10] Gorte O, Hollenbach R, Papachristou I, Steinweg C, Silve A, Frey W, Syldatk C, Ochsenreither K (2020). Evaluation of downstream processing, extraction, and quantification strategies for single cell oil produced by the oleaginous yeasts saitozyma podzolica dsm 27192 and apiotrichum porosum dsm 27194. Front Bioeng Biotechnol.

[11] Gorte O, Nazarova N, Papachristou I, Wustner R, Leber K, Syldatk C, Ochsenreither K, Frey W, Silve A (2020). Pulsed electric field treatment promotes lipid extraction on fresh oleaginous yeast saitozyma podzolica dsm 27192. Front Bioeng Biotechnol.

[12] Cao X, Xu H, Li F, Zou Y, Ran Y, Ma X, Cao Y, Xu Q, Qiao D, Cao Y (2021). One-step direct transesterification of wet yeast for biodiesel production catalyzed by magnetic nanoparticle-immobilized lipase. Renew Energy.

[13] Jin M, Slininger PJ, Dien BS, Waghmode S, Moser BR, Orjuela A, Sousa Lda C, Balan V (2015). Microbial lipid-based lignocellulosic biorefinery: feasibility and challenges. Trends Biotechnol.

[14] Zhang W, Du G, Zhou J, Chen J (2018). Regulation of sensing, transportation, and catabolism of nitrogen sources in saccharomyces cerevisiae. Microbiol Mol Biol Rev.

[15] Caron A, Richard D, Laplante M (2015). The roles of mtor complexes in lipid metabolism. Annu Rev Nutr.

[16] Gosis BS, Wada S, Thorsheim C, Li K, Jung S, Rhoades JH, Yang Y, Brandimarto J, Li L, Uehara K, Jang C, Lanza M, Sanford NB, Bornstein MR, Jeong S, Titchenell PM, Biddinger SB, Arany Z (2022). Inhibition of nonalcoholic fatty liver disease in mice by selective inhibition of mtorc1. Science.

[17] Madeira JB, Masuda CA, Maya-Monteiro CM, Matos GS, Montero-Lomeli M, Bozaquel-Morais BL (2015). Torc1 inhibition induces lipid droplet replenishment in yeast. Mol Cell Biol.

[18] Imamura S, Kawase Y (2016). Tor (target of rapamycin) is a key regulator of triacylglycerol accumulation in microalgae. Plant Signal Behav..

[19] Imamura S, Kawase Y, Kobayashi I, Sone T, Era A, Miyagishima SY, Shimojima M, Ohta H, Tanaka K (2015). Target of rapamycin (tor) plays a critical role in triacylglycerol accumulation in microalgae. Plant Mol Biol.

[20] Bracharz F, Redai V, Bach K, Qoura F, Bruck T (2017). The effects of torc signal interference on lipogenesis in the oleaginous yeast trichosporon oleaginosus. BMC Biotechnol.

[21] Teixeira V, Martins TS, Prinz WA, Costa V (2021). Target of rapamycin complex 1 (torc1), protein kinase a (pka) and cytosolic ph regulate a transcriptional circuit for lipid droplet formation. Int J Mol Sci.

[22] Conrad M, Schothorst J, Kankipati HN, Van Zeebroeck G, Rubio-Texeira M, Thevelein JM (2014). Nutrient sensing and signaling in the yeast saccharomyces cerevisiae. FEMS Microbiol Rev.

[23] Rashida Z, Srinivasan R (2021). Kog1 raptor mediates metabolic rewiring during nutrient limitation by controlling snf1 ampk activity. Sci Adv..

[24] Gross JD, Pears CJ (2021). Possible involvement of the nutrient and energy sensors mtorc1 and ampk in cell fate diversification in a non-metazoan organism. Front Cell Dev Biol.

[25] Lin SC, Hardie DG (2018). Ampk: sensing glucose as well as cellular energy status. Cell Metab.

[26] Caza M, Hu G, Price M, Perfect JR, Kronstad JW (2016). The zinc finger protein mig1 regulates mitochondrial function and azole drug susceptibility in the pathogenic fungus cryptococcus neoformans. Sphere.

[27] Seip J, Jackson R, He H, Zhu Q, Hong SP (2013). Snf1 is a regulator of lipid accumulation in yarrowia lipolytica. Appl Environ Microbiol.

[28] Wang ZP, Xu HM, Wang GY, Chi Z, Chi ZM (2013). Disruption of the mig1 gene enhances lipid biosynthesis in the oleaginous yeast yarrowia lipolytica aca-dc 50109. Biochim Biophys Acta.

[29] Gross AS, Zimmermann A, Pendl T, Schroeder S, Schoenlechner H, Knittelfelder O, Lamplmayr L, Santiso A, Aufschnaiter A, Waltenstorfer D, Ortonobes Lara S, Stryeck S, Kast C, Ruckenstuhl C, Hofer SJ, Michelitsch B, Woelflingseder M, Muller R, Carmona-Gutierrez D, Madl T, Buttner S, Frohlich KU, Shevchenko A, Eisenberg T (2019). Acetyl-coa carboxylase 1-dependent lipogenesis promotes autophagy downstream of ampk. J Biol Chem.

[30] Calvey CH, Su YK, Willis LB, McGee M, Jeffries TW (2016). Nitrogen limitation, oxygen limitation, and lipid accumulation in lipomyces starkeyi. Bioresour Technol.

[31] Hou R, Jiang C, Zheng Q, Wang C, Xu JR (2015). The area transcription factor mediates the regulation of deoxynivalenol (don) synthesis by ammonium and cyclic adenosine monophosphate (camp) signalling in fusarium graminearum. Mol Plant Pathol.

[32] Nakazawa T, Morimoto R, Wu H, Kodera R, Sakamoto M, Honda Y (2019). Dominant effects of gat1 mutations on the ligninolytic activity of the white-rot fungus pleurotus ostreatus. Fungal Biol.

[33] Song X, Wang Y, Wang P, Pu G, Zou X (2020). Gata-type transcriptional factor gat1 regulates nitrogen uptake and polymalic acid biosynthesis in polyextremotolerant fungus aureobasidium pullulans. Environ Microbiol.

[34] Klein CJL, Rasmussen JJ, Ronnow B, Olsson L, Nielsen J (1999). Investigation of the impact of mig1 and mig2 on the physiology of saccharomyces cerevisiae. J Biotechnol.

[35] Makri A, Fakas S, Aggelis G (2010). Metabolic activities of biotechnological interest in yarrowia lipolytica grown on glycerol in repeated batch cultures. Bioresour Technol.

[36] Shashkova S, Wollman AJM, Leake MC, Hohmann S (2017). The yeast mig1 transcriptional repressor is dephosphorylated by glucose-dependent and -independent mechanisms. FEMS Microbiol Lett.

[37] Alfatah M, Wong JH, Krishnan VG, Lee YC, Sin QF, Goh CJH, Kong KW, Lee WT, Lewis J, Hoon S, Arumugam P (2021). Torc1 regulates the transcriptional response to glucose and developmental cycle via the tap42-sit4-rrd1/2 pathway in saccharomyces cerevisiae. BMC Biol.

[38] Kunkel J, Luo X, Capaldi AP (2019). Integrated torc1 and pka signaling control the temporal activation of glucose-induced gene expression in yeast. Nat Commun.

[39] Kulkarni A, Buford TD, Rai R, Cooper TG (2006). Differing responses of gat1 and gln3 phosphorylation and localization to rapamycin and methionine sulfoximine treatment in saccharomyces cerevisiae. Fems Yeast Res.

[40] Pomraning KR, Bredeweg EL, Baker SE (2020). Regulation of nitrogen metabolism by gata zinc finger transcription factors in yarrowia lipolytica. mSphere.

[41] Zhang CC, Zhou CZ, Burnap RL, Peng L (2018). Carbon/nitrogen metabolic balance: Lessons from cyanobacteria. Trends Plant Sci.

[42] Jiang YL, Wang XP, Sun H, Han SJ, Li WF, Cui N, Lin GM, Zhang JY, Cheng W, Cao DD, Zhang ZY, Zhang CC, Chen Y, Zhou CZ (2018). Coordinating carbon and nitrogen metabolic signaling through the cyanobacterial global repressor ndhr. Proc Natl Acad Sci USA.

[43] Wu T, Mao XM, Kou YP, Li YL, Sun H, He YJ, Chen F (2019). Characterization of microalgal acetyl-coa synthetases with high catalytic efficiency reveals their regulatory mechanism and lipid engineering potential. J Agric Food Chem.

[44] Yan JF, Cheng RB, Lin XZ, You S, Li K, Rong H, Ma Y (2013). Overexpression of acetyl-coa synthetase increased the biomass and fatty acid proportion in microalga schizochytrium. Appl Microbiol Biotechnol.

[45] Rengel R, Smith RT, Haslam RP, Sayanova O, Vila M, Leon R (2018). Overexpression of acetyl-coa synthetase (acs) enhances the biosynthesis of neutral lipids and starch in the green microalga chlamydomonas reinhardtii. Algal Res.

[46] Plancke C, Vigeolas H, Hohner R, Roberty S, Emonds-Alt B, Larosa V, Willamme R, Duby F, Onga Dhali D, Thonart P, Hiligsmann S, Franck F, Eppe G, Cardol P, Hippler M, Remacle C (2014). Lack of isocitrate lyase in chlamydomonas leads to changes in carbon metabolism and in the response to oxidative stress under mixotrophic growth. Plant J.

[47] Bhusal RP, Jiao W, Kwai BXC, Reynisson J, Collins AJ, Sperry J, Bashiri G, Leung IKH (2019). Acetyl-coa-mediated activation of mycobacterium tuberculosis isocitrate lyase 2. Nat Commun.

[48] Eoh H, Rhee KY (2014). Methylcitrate cycle defines the bactericidal essentiality of isocitrate lyase for survival of mycobacterium tuberculosis on fatty acids. Proc Natl Acad Sci USA.

[49] Forster A, Jacobs K, Juretzek T, Mauersberger S, Barth G (2007). Overexpression of the icl1 gene changes the product ratio of citric acid production by yarrowia lipolytica. Appl Microbiol Biotechnol.

[50] Xu Y, You D, Ye BC (2021). Regx3 controls glyoxylate shunt and mycobacteria survival by directly regulating the transcription of isocitrate lyase gene in mycobacterium smegmatis. ACS Infect Dis.

[51] Cavallo E, Charreau H, Cerrutti P, Foresti ML (2017). Yarrowia lipolytica: a model yeast for citric acid production. FEMS Yeast Res.

[52] Forster A, Jacobs K, Juretzek T, Mauersberger S, Barth G (2007). Overexpression of the icl1 gene changes the product ratio of citric acid production by yarrowia lipolytica. Appl Microbiol Biotechnol..

[53] Kamzolova SV, Lunina JN, Morgunov IG (2011). Biochemistry of citric acid production from rapeseed oil by yarrowia lipolytica yeast. J Am Oil Chem Soc.

[54] Zhang YN, Wang YJ, Wang XY, Ji YQ, Cheng SZ, Wang M, Zhang C, Yu XJ, Zhao RF, Zhang WH, Jin J, Li TT, Zuo QS, Li BC (2019). Acetyl-coenzyme a acyltransferase 2 promote the differentiation of sheep precursor adipocytes into adipocytes. J Cell Biochem.

[55] Kim TY, Park H, Kim SK, Kim SJ, Park YC (2021). Production of (-)-alpha-bisabolol in metabolically engineered saccharomyces cerevisiae. J Biotechnol.

[56] Tsigie YA, Wang CY, Kasim NS, Diem QD, Huynh LH, Ho QP, Truong CT, Ju YH (2012). Oil production from yarrowia lipolytica po1g using rice bran hydrolysate. J Biomed Biotechnol..

[57] Galvez-Lopez D, Chavez-Melendez B, Vazquez-Ovando A, Rosas-Quijano R (2019). The metabolism and genetic regulation of lipids in the oleaginous yeast yarrowia lipolytica. Braz J Microbiol.

[58] Dabas N, Morschhauser J (2007). Control of ammonium permease expression and filamentous growth by the gata transcription factors gln3 and gat1 in candida albicans. Eukaryot Cell.

[59] Hunter CC, Siebert KS, Downes DJ, Wong KH, Kreutzberger SD, Fraser JA, Clarke DF, Hynes MJ, Davis MA, Todd RB (2014). Multiple nuclear localization signals mediate nuclear localization of the gata transcription factor area. Eukaryot Cell.

