# Supplementary material for: GATA-type transcriptional factor SpGAT1 interacts with SpMIG1 and promotes lipid accumulation in the oleaginous yeast Saitozyma podzolica zwy-2-3
Source: Biotechnol Biofuels Bioprod. 2022 Oct 8;15:103. doi: 10.1186/s13068-022-02177-z (PMC9548168; doi:10.1186/s13068-022-02177-z)
Supplement: Supplementary file 1 — Additional file 1: Table S1. Strains and plasmids. Table S2. Primers for gene amplification and qRT-PCR. Table S3. cDNA sequence of SpGAT1 and SpMIG1. [file 13068_2022_2177_MOESM1_ESM.docx]

**Table S1** Strains and plasmids.

| Strain/plasmid | Relevant characteristics | Source |
| --- | --- | --- |
| Strains |  |  |
| *Saitozyma podzolica* zwy-2-3 | Wild-type strain | SCTCC |
| Δ*gat1* | SpGAT1 deletion strain | This study |
| OE::*gat1* | SpGAT1 overexpression strain | This study |
| BY4741 competent cell | For subcellular localization | AngYuBio, China |
| BY4747-pYES2-SpGAT1-GFP | SpGAT1-GFP fusion expression strain | This study |
| BY4747-pYES2-SpMIG1-GFP | SpMIG1-GFP fusion expression strain | This study |
| *Agribecterium tumefaciens* AGL-1 | Used for transformation of *S. podzolica* | Biomed, China |
| *E.coli* DH5α | Host cells for plasmid preparation | Tsingke, China |
| Y1H competent cell | For yeast one-hybrid assays | WeiDiBio, China |
| Y2H competent cell | For yeast two-hybrid assays | WeiDiBio, China |
| Plasmids |  |  |
| KO-SpGAT1 | LB: upstream homologous arm, Hyg: selection marker gene, RB: downstream homologous arm | This study |
| prf-HU2-OE-SpGAT1 | pGPD:promoter, tCYC1: terminator, hyg: selection marker gene | This study |
| pYES2-SpGAT1-GFP | GFP and SpGAT1 fused expression in BY4741 | This study |
| pYES2-SpMIG1-GFP | GFP and SpMIG1 fused expression in BY4741 | This study |
| pGBKT7-SpMIG1 | For yeast two-hybrid assays | This study |
| pGADT7-SpGAT1 | For yeast two-hybrid assays or yeast one-hybrid assays | This study |
| pAbAi-promoter | For yeast one-hybrid assays | This study |

**Table S2** Primers for gene amplification and qRT-PCR.

| Name | Sequence | DNA fragment |
| --- | --- | --- |
| SpGAT1_F | ATGTCCCCATCGTACCAGA | cDNA sequence of SpGAT1 |
| SpGAT1_R | TCATGACGCCGATGACATGCT |  |
| SpMIG1_F | ATGTCCGACGACGCCG | cDNA sequence of SpMIG1 |
| SpMIG1_R | TCACGCCGCCGGATCCA |  |
| KO-LB_F | GCCAATCCTGTAGCCAGA | Amplification of LB for knockout SpGAT1 |
| KO-LB_R | atcatcttctgtcgaTCGATAGGTGTTGTAGACATCTG |  |
| KO-RB_F | gaggtaatccttcttCGTCTCGCAAGAACAGTGTCA | Amplification of RB for knockout SpGAT1 |
| KO-RB_R | GCGGTCATCGCCCATTAA |  |
| KO-HYG_F | TACAACACCTATCGAtcgacagaagatgatattga | Amplification of Hyg for knockout SpGAT1 |
| KO-HYG_R | TGTTCTTGCGAGACGaagaaggattacctctaaac |  |
| pGPD_F | CATCCGCCGATAGCATT | Amplification of pGPD promoter from genome |
| pGPD_R | TTGACACCGCAGACGAAC |  |
| OE-pGPD_F | CAGTGGTTTGGATGGCGGA | Amplification of pGPD promoter for overexpression SpGAT1 |
| OE-pGPD_R | CTGGTACGATGGGGACATTTTTGTGAGTGGGATTGG |  |
| OE-GAT1_F | CCAATCCCACTCACAAAAATGTCCCCATCGTACCAG | Amplification of SpGAT1 for overexpression SpGAT1 |
| OE-GAT1_R | GACATAACTAATTACATGATCATGACGCCGATGACAT |  |
| OE-tCYC1_F | ATGTCATCGGCGTCATGATCATGTAATTAGTTATGT | Amplification of tCYC1 terminator for overexpression SpGAT1 |
| OE-tCYC1_R | GCAAATTAAAGCCTTCGA |  |
| P1_F | CTTTTCTCTTAGGTTTACCCG | Overexpression vector validation primer |
| P1_R | TAATGCAGGAGTCGCATAAG |  |
| YZ-SpGAT1_F | GCAGTGTCCATGTCCGAGTT | Amplification full length of SpGAT1 forΔ*gat1* validation |
| YZ-SpGAT1_R | AGCGTAGATGTGAACGTGTT |  |
| YZ-pSpGAT1_F | GCTGCGACGACTTCC | Amplification partial length of SpGAT1 forΔ*gat1* validation |
| YZ-pSpGAT1_R | TGACGCCGATGACATG |  |
| YZ-Hyg+RB_F | ATGAAAAAGCCTGAACTCACCG | Amplification hyg+RB fragment |
| YZ-Hyg+RB_R | GCGGTCATCGCCCATTAA |  |
| YZ-LB+Hyg_F | GCCAATCCTGTAGCCAGA | Amplification hyg+LB fragment |
| YZ-LB+Hyg_R | TTCCTTTGCCCTCGGACGA |  |
| YZ-Hyg_F | ATGAAAAAGCCTGAACTCACCG | Amplification hyg gene fragment |
| YZ-Hyg_R | TTCCTTTGCCCTCGGACGA |  |
| qFAS1_F | TACTTCTCCGACTTTGCCTCG | *FAS1* expression level detection |
| qFAS1_R | CGCTGGTTGAACGCCTCA |  |
| qZWF1_F | GGAGGGCGTGCCTTTCAT | *ZWF1* expression level detection |
| qZWF1_R | ACCGGGTAGCTTCGAGTTCA |  |
| qG3PDH_F | GATGAGCCCACCACGAAGG | *G3PDH* expression level detection |
| qG3PDH_R | GCCAAGGGAGGAAGAAGATGA |  |
| qACC_F | TTTAGCCACAACGAGTTTACCG | *ACC* expression level detection |
| qACC_R | CGCCTGATGCCCTTTCC |  |
| qSIT4_F | GTCAGCGAACGGAGCAT | *SIT4* expression level detection |
| qSIT4_R | GGACACCGAAGGTAGCG |  |
| qSpGAT1_F | CACAAACTGCCAGACCACCA | *SpGAT1* expression level detection |
| qSpGAT1_R | GGTGAGGAGGGCTTGGACTT |  |
| qSpMIG1_F | CAACTCTGGCAAACACGG | *SpMIG1* expression level detection |
| qSpMIG1_R | GCGAAGAGGACGACACG |  |
| q18S_F | CATTAGTATTCCGTTGCTAGAGGTG | 18s reference gene |
| q18S_R | CGATCCCTAGTCGGCATAGTTTA |  |
| qDN7891_F | GCCCAGAATCATTCATCGTC | *ACAT2* expression level detection |
| qDN7891_R | TTTCCCAGCAATCCCACC |  |
| qDN7708_F | GCACCACCAACCGACAAT | *ACAT1* expression level detection |
| qDN7708_R | CACCGCATCCGACATCTT |  |
| qDN7790_F | CCGCCTCGTCCAACCTTA | *ACS1* expression level detection |
| qDN7790_R | ACCCTCCCAGTCCCATACC |  |
| qDN7275.1_F | TCTCGCCCAGTATCCCTCG | *ICL1* expression level detection |
| qDN7275.1_R | TACCACCCTTCCCTGCTCC |  |
| qDN7275.2_F | GAGGGACAGCGGATAGTGG | *ICL2* expression level detection |
| qDN7275.2_R | CGGAGATCGACCTGGAGATT |  |
| qDN7262_F | ATCACCGAAACGGGCACA | *PDC* expression level detection |
| qDN7262_R | CGACTGACCAACCGATAGAAC |  |
| pDN7891_F | cccaagcttgggTCGTGCTCGGAGGTATAGAA | Amplification promoter of *ACAT2* |
| pDN7891_R | gtcgacCGCCCTTCTTGGGTTTCA |  |
| pDN7708_F | CgagctcGGAAACCCTGGATGGAGACG | Amplification promoter of *ACAT1* |
| pDN7708_R | CGGggtaccCCGCGCTGGCTTGAGCTTGG |  |
| pDN7790_F | Cttgaattcgagctcggtaccccccacatttgggtgtctacca | Amplification promoter of *ACS1* |
| pDN7790_R | agcacatgcctcgaggtcgacCCAGAAGAGGCGGTAGGAG |  |
| pDN7275.1_F | cccaagcttgggCCAGGGGATTCGGGATA | Amplification promoter of *ICL1* |
| pDN7275.1_R | CGGggtaccCCGCGTCTGTGCGATGGATGAT |  |
| pDN7275.2_F | cttgaattcgagctcggtaccCAGATGCGTCTTTGTCCG | Amplification promoter of *ICL2* |
| pDN7275.2_R | agcacatgcctcgaggtcgacGTGCGCCAGATCCTTGA |  |
| pDN7262_F | cccaagcttgggCATCCTTGGCCCACTCG | Amplification promoter of *PDC* |
| pDN7262_R | CGGggtaccCCGCGGCGGAAACCTTGTAAT |  |

**Table S3** cDNA sequence of *SpGAT1* and *SpMIG1*.

| >SpGAT1 cDNA sequence  ATGTCCCCATCGTACCAGAACAATCGAGTCTCATTCCCTCACCCACCCAACCCGCAGTTGCCGCACCCGCAGTACATTCCCCACGGGAGGCACAGCCGAGAGGGTTCCGTCATCGGCAACGATCACTCTTCCATCGCGACTACCCCTACTCCCATGACCCCGATCGATGCGCTCGGCACGAGTGCTGGTCGTACCTACTCTGTCCCGAACGCTTTCCCTGCAGCATATGCCCGTCAGCGAGCACCGCAGATGTCTACAACACCTATCGAGCCTCCCCCGTCACCCTGGACGCTACCCGGACCCTCGGGCACCGTCACCACCCCGATCGACTGGAACAGCCCGCAAAACATGATCTGGAACCAGGGCACACCTCAGGATGACGGCACCACCGAAATCGACCCGAGCTTGTTCAACAGCTTGGCAGAGTTGATCGAGCAGAGTCAGGCGAAAGCCTCGGTCGGCGGCTCGATCAATCTGTTCGGCGCGCTGGATCCGCCTGGACCTCCCCCTCCTGGACCTATTCCCCTACACCAGCAAGCTCCGCCGCCCCCCAGTGTCGCACCCGTTGGCTCTAACCCGTCTTCCAGCCTCCTAACCCGTCGCATCCAGCAGGCTCAGCAGGGATTCAACCCCAGTCCGAATGGCGAGGCGTATTCCACCACCTCTCAATCCGTCACGCCCACTCCGACCTCCCTCCTGGCTGACCCCACCAATCTCGGCGTCCTCCCCACCCCGCCGCAGAGCTTTGGCTCTAGCTACCAGTACAACGGCGCTTCTGGACGATCCCTCAAATCGGCACCCCAGACACCGTGGCCTCTCCCGGAGAGAGCGATGGCGTACAATGAGACGCCGGTCACGACTCCCGGAGGCAGCGATTTCGGTTTTGGCAGTCCAGCTGAAGCTGGGCCTTCCAACCCGAAGCCGCATCCTCCGATCGCCTCTCGTCGGAGGGAAGCACCGCAGCCTCCAAGCTATCCCAGCTCGCTTGGCACCTCCCCGCAGAACTCGGCAGCGGCTGGGTTCAACAAGGACGGTTCTCAACAGACACCGGTCAACTGGCCTTCTGGTCTGTCACTGGCCAGCCTGCCACCCCTTCCGCCTGGTCTTTCGATCAGCCACTTGGCACAGTACGGCTCGGTCGGGCTCGAGTTGGCGATTCGTATGGGTATGGGTATCGGCATGGGTTTGGGACAGCAGGCTGGCATCGGTCTGGGTGGTCCACAGGCCAAAGGGCAAGCATTCCCGCAGCCGGGCCACAGCATCACCACGCCTTCGTCTTCGCAGCATACGTCGTCGCCCGAGGCATCGACCTCGAAGCGGGCGAGTACGGCCGGTCCTCGCACCAACATCGTGGACGATATCCTTAGCGACGATTTCTTCACCTCTCGCATCGCCTCGACACCGCTCATGACTCCTCCTATCGCCGGATCGTTCCCAACATCGCGTCGCCCCTCTCAGAGCGATGTTACCAGTCCCAACTTTCCCGAGGTGTCCTCCCCGGACGAGATGCCCAAGAACGATCCCTTGGCAACGCAGGTGTGGAAAGCCTATGCGCGGAGGAAGGAGGTGCTGCCCAACGGCCAACGTATGGAGAACCTGACCTGGCGGATGATGCACCTCACGCTCAAGAAGCAGGAGGAAGGCGGATTACCGGTGGTCAAGGAGGAGGTGAAGGATCTGTCGATCCAGTTCCCGATGGAGCTTCCCGTTCTGCCGGACACACTGCCCGAGCTGCCCGCGCAGACTCAGCAGCAGCAGCAGCAGCAGCCGGATGCGGAAGCGGAACGTGGAAGGTCCAAGGGCAAGTCTCGAGTGGTCGGATTCCAGCAGCAAAAGGAAGCGACTCCAGAGGCAGAGTCGATGGAAATGGACTGGCGTGCGGCGAGCAGGTCGCGATCCCGGATGGCCATGGATTGGCGGGCGCAATCTCGCTCCCGATCGCGGTCCGCCTTTGCAGGTCGGCCCGTCGCGTTCGCGTTCGGATCCGAGGCCCATGCACTCAACCTCCTTGCGCAGGGCTCGGATTTGCCGGCCCCAGTACCACCTGCGTACGGGCAGAGCGCGCCAGCACCCATGGCGAACTGGGCATCTGGTGGCGTCGACTTTTCGCAATCCCAGCCTGCGCCAATGCACCTCAACCTCAACAACAACCAGGGACCCAAGGACCTGTCGCAGTCTACCGGCGGGTTTGAGTTTGACCAAGCGGCACTCCGAGCGGCGGAGGCTTACGATCTGTTTGCGGCGTCGGCTCCTGCCAACGGCGCCAACGGCGCCAATGGCCCTCTGGCGCATCTCAGCATGTCCCTCGCGAGCGGTGCGAACGGTCTCGCGCAAAGCCACGGCGCGCTTCAGCCCCTAGCGCAGATGCCAATGCAAGGCCCAGGGCAGGGTCAAGGATCGGAGCAGGATCTGAGTGATCGGGCGCCAAACCTGCCTGGTATTTCCGGCCCAGGCTTGTACGCCCACACCGAGGAAAACTTCCACCCCCAATACGGTTATTTGCCCCGTCGCGTCCGCAAGACGAGCTTCGACCACACGCTCCGTCCTTTGATCGAGGAGGATCTCATGCCAACGCCCAACGGGCGCAAGCGTCAGGCTGACGCCTCTCCTCATGACGGAGCCAATGTCCCTCTGCCGGAGGGCGATACGGGCTTCCCATCTTCGGCCTTCACGTTCAACTTTCCTCAGGCGTACGAGAACTTTTTCGACATCAACGCGGCGAGCTCGTCCACGCCCGCGGCGTTTGCGATCTCGCCTACCCACAACGAGATTGGTGCTCCCACCGATGACACGACCGACTGGAGTCAGCCCGTCACGGCCGCGACATCGGCGTACGGCTCGCCCTCGGCGTTTGGGATCGACCCGTCTCTGATCCCTACGCTGCCACAGACGACGGGCGACAACCCGTTCGACTTCCAGCAGCTCATGCACCTCTATCTCAACGCCAACGCCGCGGCATCGCCGTTCACGCATATCAACCCGAGTCAAGTTCTCGGCACGGTCCCAGGTCCAGACTTCCCGTCCAACGCGGCGTCTCCGCAGAGCGTCGTCCCGACACCTCAAAGCCTGGTCCCGACTCCGCAGGGTAATGTGATCCGGCCACTACCCAAGACGGTGGGTGGCAAGCCGACCGAGTCGTCTCGCATGCCTCCTCCCCCCGCCAGGTCCAACAGCTCGCCAAACCTCCAGACCTTGAAGCTCTCCTCGGGCCCGGGCTCGAACCGCAGCTCGGCGTCGGGACACGTCAGGAATGCGTCCACTAGCGCTGCGACGACTTCCACGGGTACCACGGCGAATTCCAAGTCGTCCAAGAGCGAGAGAGCCGCCAACAAGGCCGCCGCCGCTGCCAATGCTGCTGCGGCTGCAGCGGCCGCTGCCGCTGCCAACGGCTCCGGTTCACCGCCGGGCTCGCCAAACAGCGATAACGGCGAGGGTGCGGGGTCGATCATCACCACAACGGCGAACGGAGAGTCTCCGACGGTGTGCACAAACTGCCAGACCACCAACACGCCCCTGTGGAGGAGAGACCCAGAGGGTCTCCCCCTGTGCAATGCGTGCGGGCTGTTCTATAAGCTGCACGGCGTTGTCCGACCGTTGTCGCTCAAGACAGACGTGATCAAGAAGAGGAACCGCGCCAACCCGCCCAGCAAAGAGAACGGGCCGTCTCGCAAGAACAGTGTCAGCACCAAGGCCTCCAAGTCCAAGCCCTCCTCACCTGCTACCAACGGTACTTCGGCAGGCGGCAAGAAGGCGCGGAGAGCATCGGACGGTCCGAACGTCGCGAACAACGCGAACAATGGCTCGGCCAATAACTCGGGAAGCAACTCGCTGGCGACCACGCCCACCCAGATGATCACCCCGGCGATGCTCAGCATGTCATCGGCGTCATGA |
| --- |
| >SpMIG1 cDNA sequence  ATGTCCGACGACGCCGCGGCTATCAACATCGCGTCGTTGCCCGCGCCCGTGCCCGATCCGGTCACGGGCCGGCTCGACCCCAACGACCCCGCCGTCAAGGCTCTTACCGAGGCGGCGCTCAACATGGACAAGAGCAAGATCCCCAGGCCGTACAAATGCCCTCTCTGCGATCGCGCCTTCTACCGGTTAGAGCACCAGACCCGTCACATCCGCACGCACACCGGAGAGAAGCCACACGCATGCACACATCCGGGGTGCGACAAGCGTTTCTCCCGATCCGACGAACTCACCCGTCACGCCAGGATCCATCTGCCTCCCGCGCCAGAACCCGGGGCGAAGAAGGACGAGTACGACGACCACGGCCGCCCTCACTCGATCCCCCACCTCGGACCCTCATACGGCATGGACTTTGACCGCGCCGACTACAACCCCTACTCTCTTGCCGGACTTGGCGGCTCCGCGGGCATGAACGACATCTCCGCCCTCGCCGCCGCGGCTTCGGACCAGCTGTACGAGCTCGAACGACACGAGGCCTTCCGTCGCGCCGAGTACGAGCTGCGCCACCGTCAGATCGCCGGCGCGAGGAAGAGCAACGGCAACAGCCCCAGCGGTACTCCCGGCGCCAACCTCGGCAGCGCATATGGTTTCTCCAACTCGAGCGAGCGCGACCGATTCTCGATCCACGGCATCCCTGCCCCCGGCGGAGGTCAGATGGTCTACCCGCTCTCGGCTGCTCAACCCGCCACGGCCAACCACCCCGCCGTTCCCGCCGGTACCCTCGCCGACCCGACATACCTCGTCCCCCCGACGTGCTGCCACGAGGAGTGTCACAAGAGCTACCGAAAGCGTCTCAAGGTGGCCAAGCAGACCCAGGCGTGCCCCAACTGCCTCACCATGGTACACGGCTCCAACTCGTTTGGCGGCCACGGCGGTGGCGCGGGTCACGGCCCAGGGGGCGGTAGCGGAGGCGACAGTCATCACTCGAGCAACTCGAACACGCCAAAGGACGGGAGGTCCAACATGGGTTCGCACGATGATCTCACCAAGCTGGGCAACGGTGGAGGAGGCTCGCTCGGGAACTCTTACAACCTCCACCATCACCACGCGCAGATCTCGCAGCAGCTTGCCAGGCTTCAGCAGCAGCATCAGCTGGCGCTGCAAAAGGCGCGTCAGCAGTCGCAGATGGCTGGAGCCGGCGCGGGTCCGTCTCGTCACCTCAAGCCGTACACGCTCGATCTCCACGCCCACCGCGGCCTGATCCCGTCGACTCAGGTGTCGGCCGCGCCCAGCCCCGCGTCGAGCGACAGCGAGGACGACGACGAAGGCATGGTCGGCAACGCGAGCTTCGAATCGTTCCTGCCAGCCACGAGCCCCGTCTTGAGCGGCATGCGCCACATGAGCCTGTTCCAGCAGGGCAAAGCGATGACCGCGCCTGCTTCGGCGGTCACCAGCCCTGTGCACTCGCGCAACCCTTCGCGCGCGGGCTCGCCTGTCGAAGGTCACTCTGCCAACTCTGGCAAACACGGCCACGGTTCTCATCGCGCTCACGACGCCAAGACGCGCAGTCACCCGTACACGCATTTTTCAGCGACGACTCCCAACTCGCCCCATTTCCCGCCGACAAAGTCCCGCGGGATGTCTCCTCCCAAGCTCGCGCGGACGCTGTCCAACTCGCACGGCGTGTCGTCCTCTTCGCACCACTCGAGCGCTGGGGCCAGGCAGTCGGTCGAGGATATCCTCAACGCGCACGCGATCCCACCCCCGCCACCGCCTTCTGACCGGACTCTGCCGCCTCCGAACTCGTCCGCGTCATTCACCTCGTCGGTACCGTCGGTGTCGTACTCGCTGTCGTCGCAACCGACCTCGGCTCACGCCTCGCCTGCTACGTCGCGAGCCGGCTCGCCAGTGCACAGCACGCACAGCACGAACCCGGCCCACGCGTCGCACCACCATCTCGCGCACTCTGTCCGCGCGGCGTTTGGCATGACTCCCATGGGCATGGCGATGGCCGCGGGCGGTGGCGGTGGCGCGGGAGGCGTCGCGTCGTCTATCTCGCCGAAATCGGGCGTGATCTCCCCTCCGAGGAGGCTCGCGCCGATGAACGGTGGGGACGGGGTGAAGCATTTGCCGAGTTCTCAAGGGGCACTAGCCCGGTGATGATGGGGATGGATGTGGATCCGGCGGCGTGA |
