# Supplementary material for: GATA-type transcriptional factor SpGAT1 interacts with SpMIG1 and promotes lipid accumulation in the oleaginous yeast Saitozyma podzolica zwy-2-3
Source: Biotechnol Biofuels Bioprod. 2022 Oct 8;15:103. doi: 10.1186/s13068-022-02177-z (PMC9548168; doi:10.1186/s13068-022-02177-z)
Supplement: Supplementary file 2 — Additional file 2: Fig. S1. Single cell lipid yield and fatty acid profile of S.podzolica zwy-2-3 cultivated in rapamycin treatment and different C/N ratio mediums. a Single cell lipid yield stained by Nile red. b Fatty acid profiles analyzed by GC-MS. Fig. S2. Results of SpGAT1 mutant identification. a Results of amplifying hygromycin gene, homologous arm, and part of SpGAT1 gene (lane 1: marker 5000 bp, lane 2: amplifying hygromycin gene and LB homologous arm in WT, lane 3: amplifying hygromycin gene and LB homologous arm in Δgat1, lane 4: amplifying hygromycin gene and RB homologous arm in WT, lane 5: amplifying hygromycin gene plus RB homologous arm in Δgat1, lane 6: amplifying part of SpGAT1 gene in Δgat1, lane 7: amplifying part of SpGAT1 gene in WT). b Results of amplifying full length of SpGAT1 (lane 1: marker 2000 bp, lane 2: WT, lane 3: Δgat1). c Results of amplifying hygromycin gene by colony PCR (lane 1: marker 5000 bp, lane2: OE::gat1, lane 3: WT). d Detecting SpGAT1 expression level in WT and OE::gat1 by qRT-PCR. Fig. S3. Effects of SpGAT1 on fatty acid profiles and different carbon/nitrogen sources utilization in S.podzolica zwy-2-3. a Fatty acid profiles in WT, Δgat1 and OE::gat1 cultivated in low and high C/N ratio mediums. b Utilization different nitrogen sources in WT, Δgat1, and OE::gat1. c Utilization different carbon sources in WT, Δgat1, and OE::gat1. Fig. S4. Results of subcellular localization observed by fluorescence microscopy. Cyto: transcription factors were cytoplasmic localization, Nuclear: transcription factors were nuclear localization, Nucl-Cyto: transcription factors were located in cytoplasm and nuclear. Fig. S5. Prediction of SpGAT1 interacting protein. a Predicted by GeneMANIA database, b Predicted by String database. [file 13068_2022_2177_MOESM2_ESM.docx]

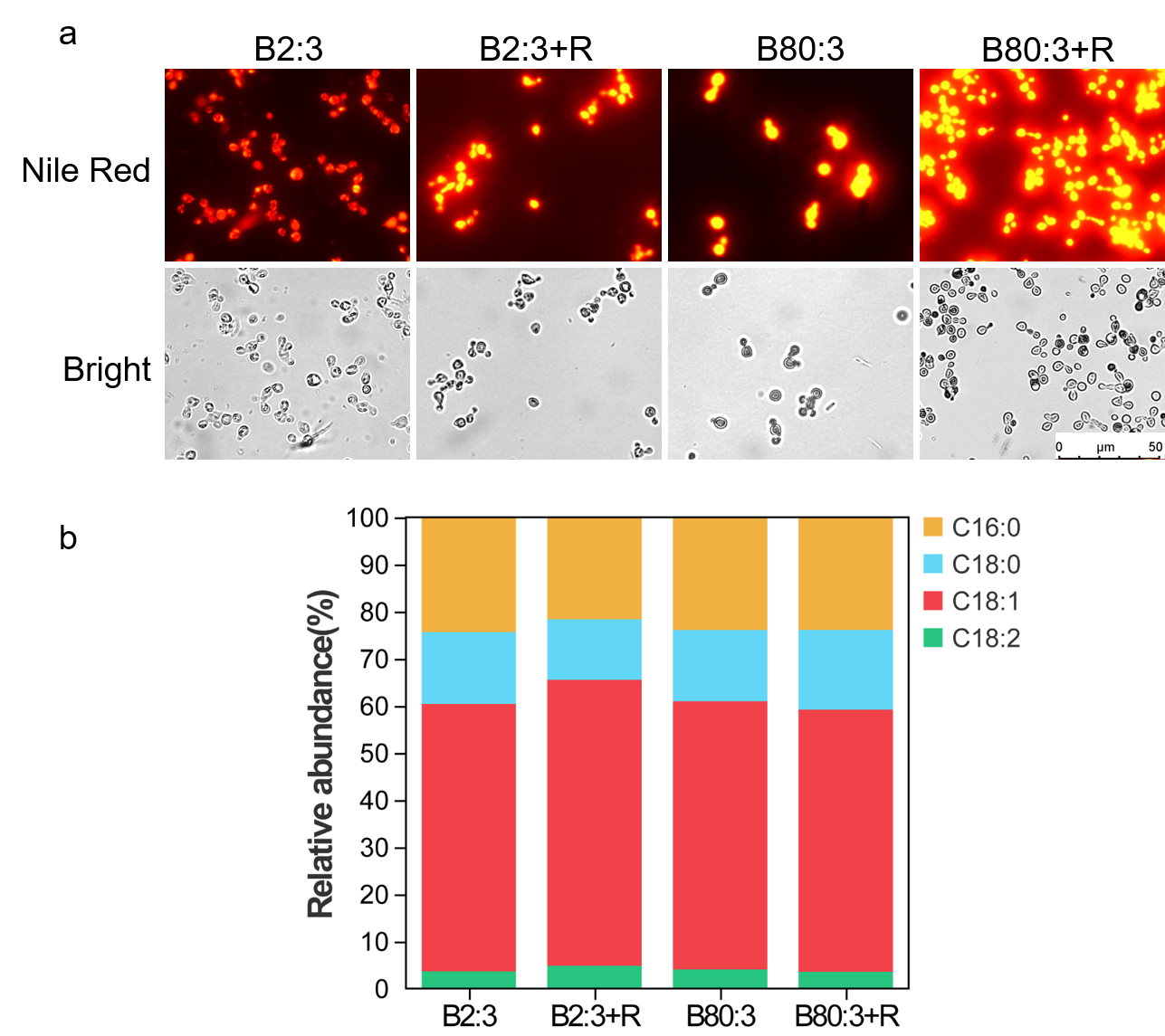


**Fig. S1** Single cell lipid yield and fatty acid profile of *S.podzolica* zwy-2-3 cultivated in rapamycin treatment and different C/N ratio mediums. **a** Single cell lipid yield stained by Nile red. **b** Fatty acid profiles analyzed by GC-MS.


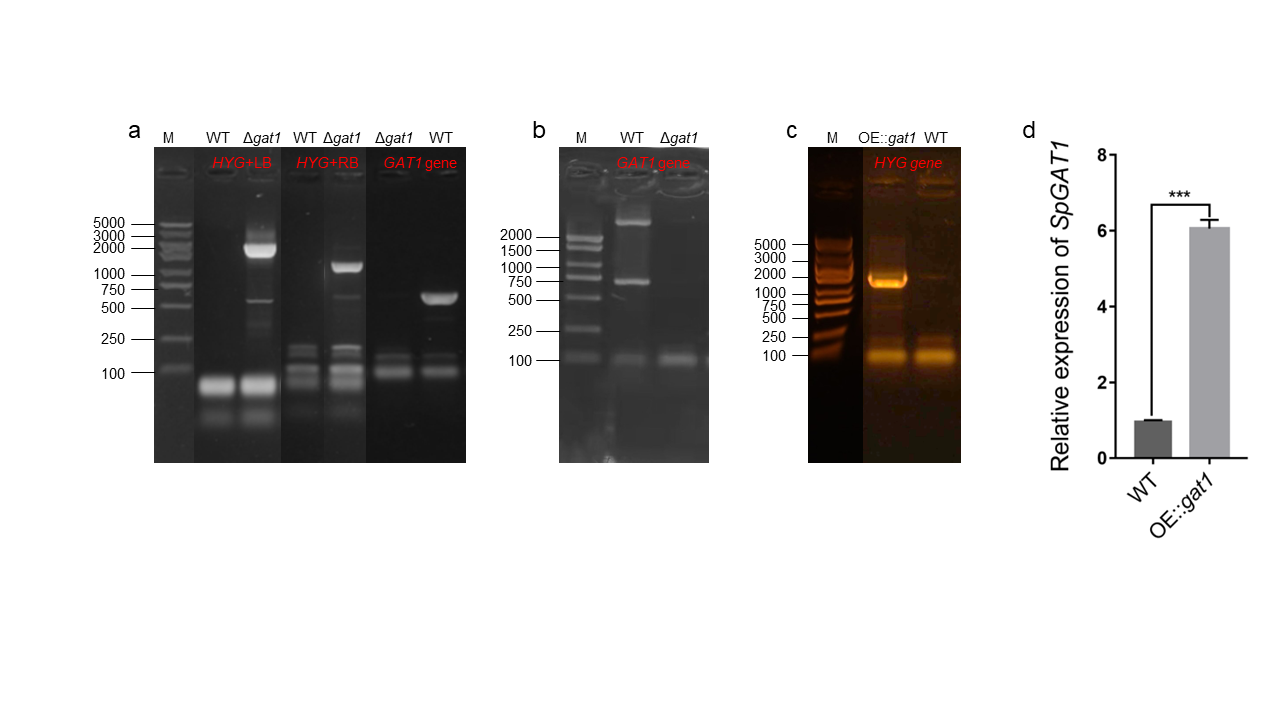


**Fig. S2** Results of *SpGAT1* mutant identification. **a** Results of amplifying hygromycin gene, homologous arm, and part of *SpGAT1* gene (lane 1: marker 5000 bp, lane 2: amplifying hygromycin gene and LB homologous arm in WT, lane 3: amplifying hygromycin gene and LB homologous arm in Δ*gat1*, lane 4: amplifying hygromycin gene and RB homologous arm in WT, lane 5: amplifying hygromycin gene plus RB homologous arm in Δ*gat1*, lane 6: amplifying part of *SpGAT1* gene in Δ*gat1*, lane 7: amplifying part of *SpGAT1* gene in WT). **b** Results of amplifying full length of *SpGAT1* (lane 1: marker 2000 bp, lane 2: WT, lane 3: Δ*gat1*). **c** Results of amplifying hygromycin gene by colony PCR (lane 1: marker 5000 bp, lane2: OE::*gat1*, lane 3: WT). **d** Detecting *SpGAT1* expression level in WT and OE::*gat1* by qRT-PCR.

**
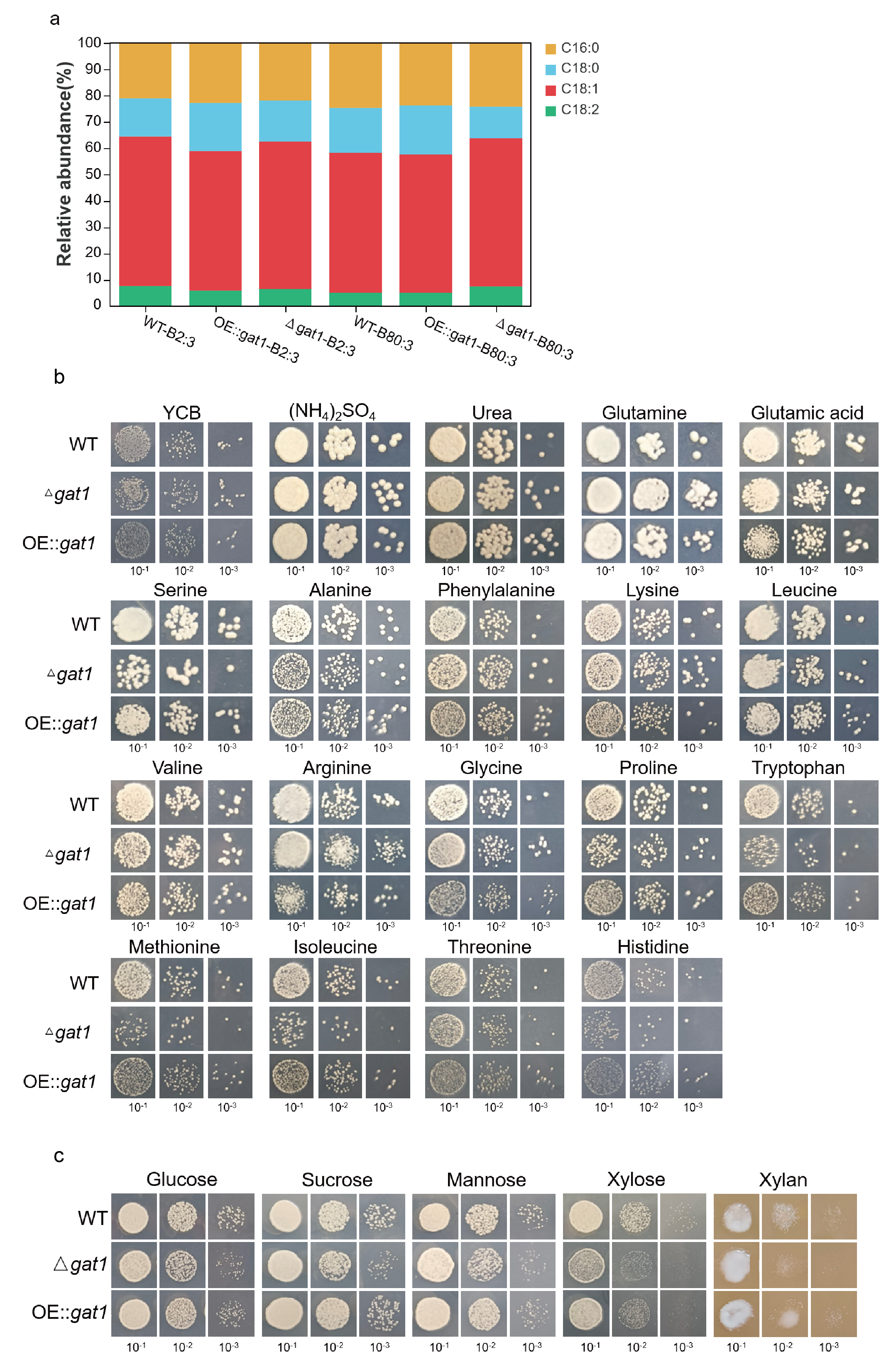
**

**Fig. S3** Effects of SpGAT1 on fatty acid profiles and different carbon/nitrogen sources utilization in *S.podzolica* zwy-2-3. **a** Fatty acid profiles in WT, Δ*gat1* and OE::*gat1* cultivated in low and high C/N ratio mediums. **b** Utilization different nitrogen sources in WT, Δ*gat1*, and OE::*gat1*. **c** Utilization different carbon sources in WT, Δ*gat1*, and OE::*gat1*.


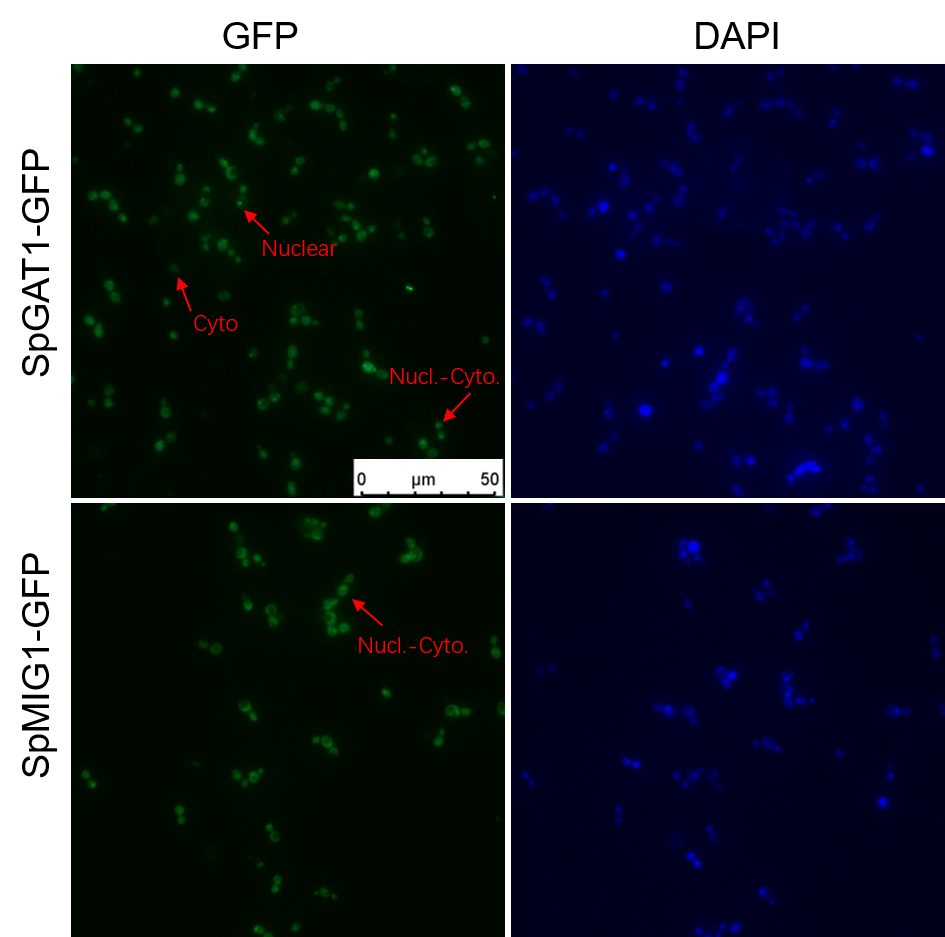


**Fig. S4** Results of subcellular localization observed by fluorescence microscopy. Cyto: transcription factors were cytoplasmic localization, Nuclear: transcription factors were nuclear localization, Nucl-Cyto: transcription factors were located in cytoplasm and nuclear.


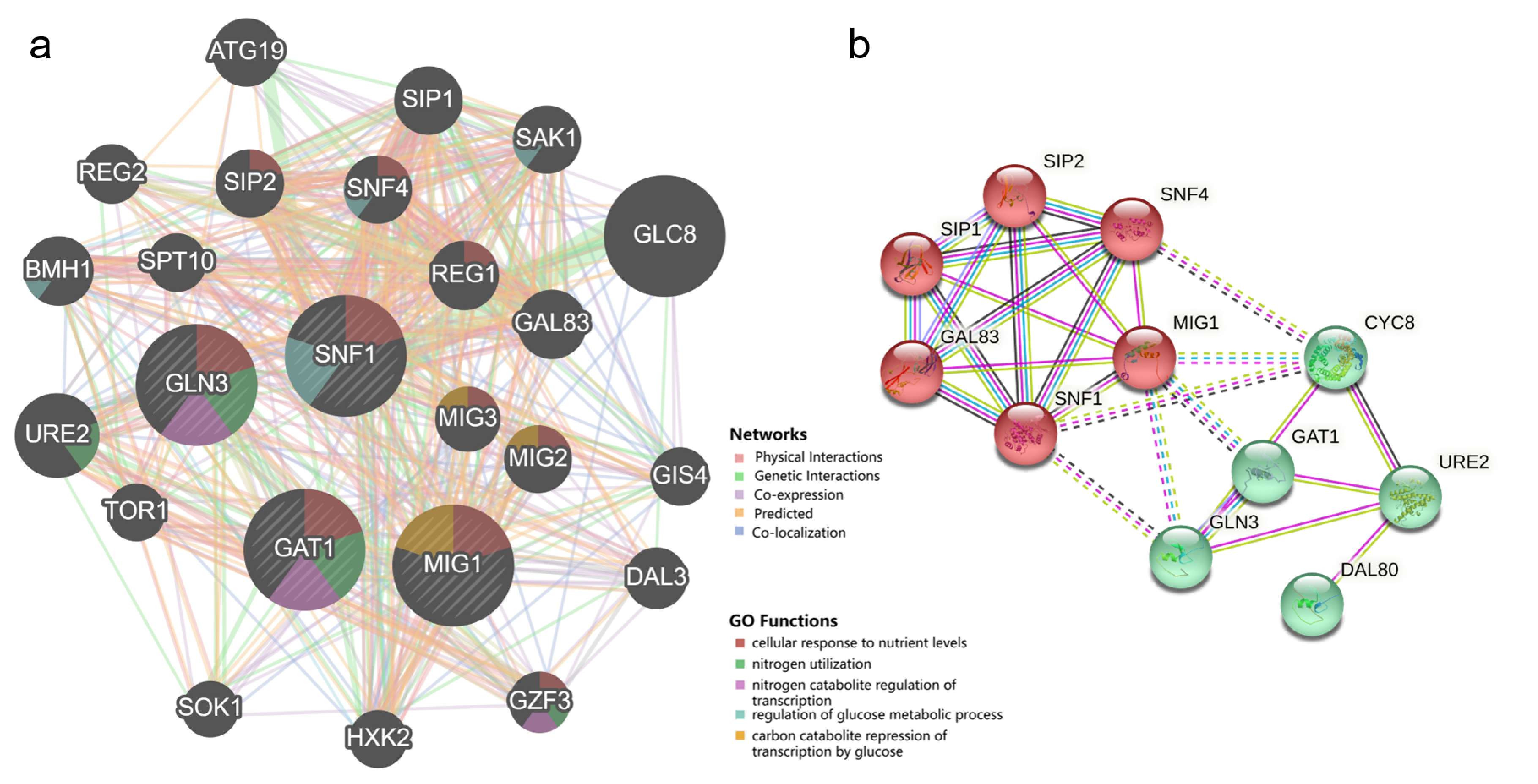


**Fig. S5** Prediction of SpGAT1 interacting protein. **a** Predicted by GeneMANIA database, **b** Predicted by String database.
